# Polymorphisms in Thioredoxin Reductase and Selenoprotein K Genes and Selenium Status Modulate Risk of Prostate Cancer

**DOI:** 10.1371/journal.pone.0048709

**Published:** 2012-11-01

**Authors:** Catherine Méplan, Sabine Rohrmann, Astrid Steinbrecher, Lutz Schomburg, Eugène Jansen, Jakob Linseisen, John Hesketh

**Affiliations:** 1 Institute for Cell and Molecular Biosciences, Newcastle University, Newcastle-upon-Tyne, United Kingdom; 2 Human Nutrition Research Centre, Newcastle University, Newcastle-upon-Tyne, United Kingdom; 3 Division of Cancer Epidemiology and Prevention, Institute of Social and Preventive Medicine, University of Zurich, Zurich, Switzerland; 4 Division of Cancer Epidemiology, German Cancer Research Center, Heidelberg, Germany; 5 Institute for Experimental Endocrinology, Charité - University Medicine Berlin, Berlin, Germany; 6 Laboratory for Health Protection Research, National Institute for Public Health and the Environment, Bilthoven, The Netherlands; 7 Institute of Epidemiology, Helmholtz Zentrum München, Neuherberg, Germany; The University of Hong Kong, China

## Abstract

Increased dietary intake of Selenium (Se) has been suggested to lower prostate cancer mortality, but supplementation trials have produced conflicting results. Se is incorporated into 25 selenoproteins. The aim of this work was to assess whether risk of prostate cancer is affected by genetic variants in genes coding for selenoproteins, either alone or in combination with Se status. 248 cases and 492 controls from an EPIC-Heidelberg nested case-control study were subjected to two-stage genotyping with an initial screening phase in which 384 tagging-SNPs covering 72 Se-related genes were determined in 94 cases and 94 controls using the Illumina Goldengate methodology. This analysis was followed by a second phase in which genotyping for candidate SNPs identified in the first phase was carried out in the full study using Sequenom. Risk of high-grade or advanced stage prostate cancer was modified by interactions between serum markers of Se status and genotypes for rs9880056 in *SELK*, rs9605030 and rs9605031 in *TXNRD2*, and rs7310505 in *TXNRD1*. No significant effects of SNPs on prostate cancer risk were observed when grade or Se status was not taken into account. In conclusion, the risk of high-grade or advanced-stage prostate cancer is significantly altered by a combination of genotype for SNPs in selenoprotein genes and Se status. The findings contribute to explaining the biological effects of selenium intake and genetic factors in prostate cancer development and highlight potential roles of thioredoxin reductases and selenoprotein K in tumour progression.

## Introduction

The micronutrient Selenium (Se) is essential for human health and sub-optimal intake has been suggested to increase risk of various multifactorial diseases [1], [2]. Increased dietary intake of Se has been proposed to lower cancer mortality [3] and in particular Se has been reported to have a protective effect against prostate cancer [4], based partly on the results of a trial in the US that found an additional 200 µg Se/day to lower prostate cancer incidence in individuals who had relatively low Se status prior to supplementation [5]. However, a second supplementation trial (SELECT) failed to confirm this observation [6]. Although the different outcomes of these trials are likely to be due to a higher baseline Se status in the more recent SELECT study [7], they may also be affected by differences in the characteristics of the probands, such as pattern and prevalence of Se-related genetic variants in the study cohorts.

The biological functions of Se are carried out primarily by selenoproteins which contain Se in the form of the amino acid selenocysteine [8] and it is likely that the anti-carcinogenic properties of Se are brought about through these selenoproteins [9]. The selenoproteins have functions in cellular antioxidant protection (glutathione peroxidases, selenoproteins W and H), redox control (thioredoxin reductases), Se transport (selenoprotein P), and the endoplasmic reticulum unfolded protein response (selenoprotein S, 15 kDa selenoprotein, selenoprotein K) [10]. GPx3 and selenoprotein P (SePP) are secreted into the bloodstream and their plasma level, as well as serum Se, are commonly used as markers of Se status [11], [12]. A functional interaction between selenoproteins and prostate cancer has been reported, i.e. serum Se and selenoprotein P (SePP) concentrations are reduced in prostate cancer patients and this is correlated with disease severity [13]. This in turn could reduce selenoprotein expression and associated anti-oxidant defense resulting in increased oxidative damage leading to prostate cancer progression [14].

Selenocysteine incorporation into selenoproteins occurs during translation and requires proteins such as SECIS-binding protein 2 (SBP2) [7], [10]. Genetic variants in genes encoding the selenoproteins or components of the selenocysteine incorporation machinery would be expected to influence the biological pathways that are modulated by selenoproteins [15], [16]. Indeed, functional single nucleotide polymorphisms (SNPs) have been identified in a number of selenoprotein genes [13], [14] and disease association studies have linked variants in *SEPP1*, *GPX1*, *GPX4*, *SEP15* or *SELS* to risk of various diseases [15], [16]. Three recent studies have studied the association of single selenoprotein gene variants with prostate cancer risk [13], [17], [18] and importantly they suggest that interactions between different SNPs in selenoprotein genes or antioxidant protein genes and Se status may influence susceptibility to prostate cancer or disease mortality. The common mechanism by which Se is incorporated into selenoproteins, the hierarchy of selenoprotein synthesis when Se supply is limited and the related functions of several selenoproteins (e.g. in redox control and unfolded protein response) all emphasise that selenoproteins are components of an integrated metabolic pathway. This close relationship between selenoproteins suggests that the influence of genetics on selenoprotein function and related disease risk is complex involving multiple interacting variants and both genetic and nutritional factors. However, such a comprehensive study of SNPs throughout the “Se pathway” in relation to prostate cancer has not been carried out.

As the selenoprotein family and selenoprotein biosynthesis pathway are well characterised, the aim of the present study was to investigate the association between SNPs throughout the genes encoding selenoproteins, factors essential for selenocysteine incorporation and related antioxidant proteins, Se status (as assessed by measurement of total serum Se, selenoprotein P (SePP) concentration and serum glutathione peroxidase (GPx3) activity) and prostate cancer risk in a European population with a Se status lower than that found in the USA. To achieve this aim, DNA samples from EPIC-Heidelberg, a prospective cohort study aiming to evaluate the association between dietary, lifestyle and metabolic factors and the risk of cancer, were genotyped and plasma samples analysed for plasma selenium status, selenoprotein P concentration and GPx activity. Previously, the samples had been analysed for six selenoprotein SNPs and rs1053040 in *GPX1* was found to modulate the effect of serum Se on prostate cancer risk [13]. These six SNPs were not examined in the present study but instead the approach taken was a two-stage genotyping study: the first stage was an unbiased hypothesis-generating phase in which genotyping for 384 tagging-SNPs covering 72 Se-related genes (including selenoprotein genes, selenoprotein synthesis machinery, factors known to be influenced by selenoproteins, some related transcription factors and genes in linkage disequilibrium with these genes) was carried out in 94 advanced prostate cancer cases and an equal number of matched controls; in the second phase genotyping for a selected number of SNPs identified in the first phase was carried out in all 492 cases and controls.

## Methods

### Study Population and Data Assessment

The EPIC-Heidelberg study was designed to evaluate the association between dietary, lifestyle and metabolic factors and the risk of cancer. A random sample of the general population of Heidelberg, Germany, and surrounding communities was provided by the local registries and invited to participate. From 1994 to 1998, 11928 men (aged 40–64) and 13612 women (aged 35–64) were recruited, comprising 38% of those approached [19]. Details of the collection of dietary, lifestyle and socioeconomic data have been described previously [13]. Blood samples were taken and fractionated into serum, plasma, buffy coat and erythrocytes, and stored stored in liquid nitrogen. All participants gave written informed consent and the study was approved by the ethics committee of the Heidelberg Medical School. Subsequently participants were contacted three times (every 2–3 years) by follow-up questionnaires to assess health status; participation rates of the completed three follow-ups were >90%. Based on all male EPIC-Heidelberg participants with blood samples available and free of prevalent cancer (except non-melanoma skin cancer) at baseline we set up a nested case-control study. Incident prostate cancer cases diagnosed by end of February 2007 were selected and two controls were matched per case by age (5-year age groups) and time of recruitment (6 month intervals) following an incidence density matching protocol. The final study comprised 248 cases and 492 controls.

Self-reported cases of prostate cancer were verified by examination of medical records or death certificates (C61, C63.8 and C63.9; International Classification of Diseases for Oncology, 2^nd^ edition). Tumor grade information (Gleason histologic grade) was used to categorize cases as high-grade (Gleason score ≥7), low-grade (<7) or unknown. Advanced prostate cancer was defined as prostate cancer with a Gleason sum score ≥7, TNM staging score of T3/4, N1-3 or M1 or prostate cancer as underlying cause of death. During the 2^nd^ and 3^rd^ follow-up rounds questions addressed history of prostate cancer in 1^st^ degree relatives and participation in prostate specific antigen (PSA) screening. Only those cases who participated in screening before the date of cancer diagnosis were coded as having a positive screening history. Similarly, only controls participating in screening before the date of diagnosis were classified as controls participating in prostate cancer screening. Samples for analysis during the initial screening phase of genotyping include advanced prostate cancer cases and one matched control per case.

### Genotyping

Genomic DNA was extracted from buffy coat with FlexiGene Kit (Qiagen, Hilden, Germany) in accordance with the manufacturer’s instructions. DNA was stored at 4°C until use. A custom Illumina™ GoldenGate assay was designed for analysis of 384 candidate SNPs (tagSNPs and potential functional SNPs) in the selenoprotein and selenium pathway. Tag SNPs were selected, using Haploview 3.2, with a cutoff minimum minor allele frequency (MAF, in CEU population) of 0.05 and pairwise tagging (r^2^ = 1-0.8). To include promoter regions and SNPs in LD in neighboring genes, regions covering the coding region +/−2 to15 kbp beyond the 5′ and 3′ ends were used for the selection. Selected SNPs were then assessed for suitability for the Illumina™ GoldenGate genotyping platform, and the analysis was carried out on SNPs which were GoldeneGate validated or two-hit validated with scores >60%. The average call rate was >99%. The list of SNPs on the chip is presented in Table S1. Genotyping using the custom chip was carried out by ServiceXs, Leiden, The Netherlands.

Subsequently genotyping for selected SNPs (rs9880056 in *SELK*, rs7310505 in *TXNRD1*, rs9605031 and rs9605030 in *TXNRD2*, rs28665122 in *SEPS1* and rs3211684 in *SBP2*) was performed as multiplex on the MassArray® system (Sequenom, San Diego, USA) applying the iPLEX® method and MALDI-TOF mass spectrometry for analyte detection. The analysis was carried out by Bioglobe (Hamburg, Germany). All duplicated samples (quality control repeats of 8% of the samples) to verify inter-experimental reproducibility and accuracy delivered concordant genotype results. Similarly a control sample applied on every plate yielded identical genotypes. All laboratory analyses were carried out with the laboratory personnel blinded to the case-control status.

### GPx Activity, Serum Se and Selenoprotein P Concentrations

GPx activity was determined with Ransel RS 505 kits (Randox, Crumlin, UK), as described previously [13], [20]. Total serum Se was determined by dynamic reaction cell-inductively coupled plasma field mass spectrometry as described previously [13], [21]. Coefficients of intra-assay and inter-assay variation have been reported previously [13]. Serum Selenoprotein P concentration was measured by an immunoluminometric sandwich assay [22] as described in detail previously [13]. Six samples had insufficient amounts of serum to be analyzed.

### Statistical Analysis

Baseline characteristics of the study population are given as mean and standard deviation or percentages by case-control status. Serum Se and SePP concentrations as well as GPx3 activity were nearly normally distributed and are presented as mean and standard deviation.

Among healthy controls, Pearson correlation coefficients were computed for serum Se, SePP and GPx3 activity. Genotype frequencies for the selected polymorphisms were computed and deviations from Hardy-Weinberg equilibrium were determined by Chi^2^ test. Conditional logistic regression stratified by the matched case set was used to calculate odds ratios (OR) and 95% confidence intervals (CI) for the association of the SNPs with prostate cancer risk, using the frequent homozygous genotype wild type as reference category. As reported earlier [13], the final model included participation in PSA screening, smoking status, vigorous physical; activity and family history of prostate cancer as adjustments.

To evaluate potential effect modification of the association between serum Se concentration and prostate cancer risk by genotype, we calculated OR (and 95% CI) of prostate cancer for the continuous variables (serum Se, SePP and GPx) stratified by genotype with unconditional logistic regression adjusting for the matching variables (time of recruitment and 5-year age group). Additionally adjustments were made for family history of prostate cancer, participation in PSA screening, smoking status and vigorous physical activity. Due to small numbers in the homozygous mutant genotype we also combined the heterozygote and homozygote (mutant) categories. We tested for interaction by comparing the unconditional logistic regression model with and without crossproduct terms (of genotype and continuous Se variable) based on the likelihood ratio statistic. This analysis was repeated in the subgroups according to stage and grade of prostate cancer. All analyses were performed with SAS 9.1 (SAS Institute, Cary, USA).

## Results

Baseline characteristics of the study population have been presented previously [13]. Cases and controls were matched for age and were of comparable BMI. No significant differences were observed in mean serum concentration of Se or SePP or GPx3 activity between cases and controls [13].

### Identification of SNPs Interacting with Se Status Markers to Determine Prostate Cancer Risk

Genotyping was carried out in two phases. In the initial hypothesis-generating phase the sub-group of advanced prostate cancer cases (n = 94) and an equal number of matched controls were genotyped for 384 SNPs including functional SNPs in selenoprotein genes and tagging SNPs covering the 25 selenoprotein genes and factors important for selenoprotein biosynthesis (Table S1) selected as described in Materials and Methods. Genotype alone showed no significant effects of selenoprotein SNPs on prostate cancer risk. However, taking both genotype and Se status into account, analysis of all these variants for the 94 advanced prostate cancer cases and controls indicated that a limited number of SNPs showed a significant interaction with one or more measures of Se status on prostate cancer risk when the SNP was considered as either a continuous variable or in a dominant mode (data not shown). To limit the number of tests on the whole cohort SNPs were identified which showed statistically significant interaction with at least one measure of Se status on prostate cancer risk at the 1% level. Using these criteria, rs9880056 in *SELK*, rs9605030 and rs9605031 in *TXNRD2*, rs7310505 in *TXNRD1*, rs3211684 in *SBP2* and rs28665122 in *SEPS1* were considered for further study.

### Interaction between Genotype and Selenium Status Affects Prostate Cancer Risk for Selected Individual SNPs

In a second genotyping phase, the full nested case-control study (248 cases/492 controls) was genotyped for SNPs that showed a significant interaction with markers of Se status in the first, pathway analysis, stage (see above: SNPs in *TXNRD1*, *TXNRD2*, *SELK*, *SEPS1* and *SBP2*). The genotype frequencies are shown in Table 1. In the controls, genotype frequencies of these five SNPs were in Hardy-Weinberg equilibrium. There was no statistically significant main effect of any of these five SNPs on prostate cancer risk. However, as shown in Table 2, when the influence of Se status on the effects of genotype for the selected polymorphisms was taken into account SNPs in *TXNRD1* and *TXNRD2* genes were found to influence prostate cancer risk. Firstly, subjects homozygous for the C allele of rs7310505 in *TXNRD1* had an OR of 0.88 for prostate cancer risk (95% CI = 0.78–1.00, p = 0.045) per 10 µg/l increase in serum Se concentration, whereas in subjects carrying the A allele (heterozygous or homozygous) no association was found (P_interaction_ = 0.06). Secondly, subjects carrying the T allele (heterozygous or homozygous) for rs9605031 in the *TXNRD2* gene had an OR of 0.82 for disease risk (95% CI = 0.69–0.96, p = 0.016) per 10 µg/l increase in serum Se concentration, whereas in subjects carrying the homozygous C no association was found (P_interaction_ = 0.02). Interaction terms with other markers of Se status (serum SePP or GPx activity) did not reveal significant changes in prostate cancer risk.

**Table 1:** Genotype frequencies of polymorphisms in selected selenoproteins association with prostate cancer risk in the EPIC-Heidelberg nested case-control study.

Gene	RS number	Genotype	Control N	Case N	OR (95%CI)	p-value
*SELK*	rs9880056	TT	256	122		
		TC	207	105	1.07 (0.78, 1.47)	0.68
		CC	29	21	1.51 (0.83, 2.77)	0.18
		TC/CC	236	126	1.12 (0.83, 1.52)	0.47
*TXNRD2_1*	rs9605030	CC	377	184		
		CT	104	61	1.18 (0.83, 1.68)	0.36
		TT	11	3	0.54 (0.15, 1.97)	0.35
		CT/TT	115	64	1.13 (0.80, 1.60)	0.49
*TXNRD2_2*	rs9605031	CC	272	128		
		CT	185	98	1.12 (0.82, 1.53)	0.48
		TT	35	22	1.32 (0.75, 2.33)	0.34
		CT/TT	220	120	1.15 (0.85, 1.55)	0.36
*SBP2*	rs3211684	TT	436	218		
		GT	54	28	1.03 (0.65, 1.66)	0.89
		GG	2	2	2.01 (0.28, 14.27)	0.49
		GT/GG	56	30	1.07 (0.67, 1.69)	0.78
*TXNRD1*	rs7310505	CC	310	149		
		CA	161	88	1.14 (0.82, 1.57)	0.43
		AA	21	11	1.09 (0.51, 2.32)	0.83
		CA/AA	182	99	1.13 (0.83, 1.54)	0.43
*SEPS1*	rs28665122	CC	365	192		
		CT	121	53	0.84 (0.59, 1.20)	0.34
		TT	6	3	0.98 (0.25, 3.93)	0.98
		CT/TT	127	56	0.85 (0.60, 1.20)	0.35

OR, 95% confidence interval (CI) and P values were calculated for each SNP analysed using logistic regression. For each SNP, ORs are presented with reference to the most frequent homozygous genotype.

**Table 2:** Odds ratio and 95% confidence interval for association of serum Se concentration, serum SePP concentration or serum GPx activity and prostate cancer in strata of genetic polymorphisms.

Gene	RS number	Genotype	Cases/Controls	OR (95% CI)	p-value
*SELK*	rs9880056				
		TT	121/254	0.94 (0.81, 1.07)	0.34
		TC/CC	123/236	0.89 (0.77, 1.02)	0.09
	***P_interaction_ serum Se***				0.46
*SELK*	rs9880056				
		TT	121/255	0.89 (0.61, 1.28)	0.52
		TC/CC	126/236	0.78 (0.54, 1.12)	0.17
	***P_interaction_ serum SePP***				0.63
*SELK*	rs9880056				
		TT	122/256	0.93 (0.79, 1.10)	0.39
		TC/CC	126/236	0.93 (0.79, 1.09)	0.36
	***P_interaction_ serum GPx activity***				0.97
*TXNRD 2_1*	rs9605030				
		CC	182/377	0.95 (0.85, 1.06)	0.39
		CT/TT	62/113	0.86 (0.69, 1.08)	0.19
	***P_interaction_ serum Se***				0.18
*TXNRD2_1*	rs9605030				
		CC	184/376	0.87 (0.65, 1.16)	0.34
		CT/TT	63/115	0.85 (0.49, 1.48)	0.57
	***P_interaction_ serum SePP***				0.9
*TXNRD2_1*	rs9605030				
		CC	184/377	0.92 (0.80, 1.05)	0.21
		CT/TT	64/115	0.96 (0.77, 1.19)	0.72
	***P_interaction_ serum GPx activity***				0.44
*TXNRD2_2*	rs9605031				
		CC	127/272	0.99 (0.88, 1.12)	0.90
		CT/TT	117/218	0.82 (0.69, 0.96)	***0.02***
	***P_interaction_ serum Se***				***0.02***
*TXNRD2_2*	rs9605031				
		CC	128/271	0.91 (0.66, 1.27)	0.58
		CT/TT	119/220	0.75 (0.49, 1.14)	0.18
	***P_interaction_ serum SePP***				0.58
*TXNRD2_2*	rs9605031				
		CC	128/272	0.95 (0.81, 1.11)	0.52
		CT/TT	120/220	0.93 (0.79, 1.10)	0.40
	***P_interaction_ serum GPx activity***				0.93
*SBP2*	rs3211684				
		GT/GG	29/56	0.97 (0.65, 1.44)	0.87
		TT	215/434	0.92 (0.83, 1.02)	0.12
	***P_interaction_ serum Se***				0.65
*SBP2*	rs3211684				
		GT/GG	30/56	0.92 (0.32, 2.62)	0.87
		TT	217/435	0.84 (0.64, 1.10)	0.20
	***P_interaction_ serum SePP***				0.51
*SBP2*	rs3211684				
		GT/GG	30/56	0.99 (0.64, 1.55)	0.971
		TT	218/436	0.93 (0.83, 1.05)	0.27
	P***_interaction_*** serum GPx activity				0.66
*TXNRD1*	rs7310505				
		CC	146/310	0.88 (0.78, 1.00)	***0.04***
		CA/AA	98/180	1.03 (0.87, 1.22)	0.72
	***P_interaction_ serum Se***				0.06
*TXNRD1*	rs7310505				
		CC	149/310	0.84 (0.61, 1.14)	0.26
		CA/AA	98/181	0.98 (0.62, 1.57)	0.95
	***P_interaction_ serum SePP***				0.47
*TXNRD1*	rs7310505				
		CC	149/310	0.89 (0.77, 1.04)	0.15
		CA/AA	99/182	1 (0.84, 1.19)	0.97
	***P_interaction_ serum GPx activity***				0.16
*SEPS1*	rs28665122				
		CC	188/363	0.95 (0.85, 1.06)	0.33
		CT/TT	56/127	0.86 (0.68, 1.08)	0.19
	***P_interaction_ serum Se***				0.11
*SEPS1*	rs28665122				
		CC	191/365	0.87 (0.66, 1.17)	0.36
		CT/TT	56/126	0.73 (0.41, 1.29)	0.27
	***P_interaction_ serum SePP***				0.3
*SEPS1*	rs28665122				
		CC	192/365	0.97 (0.85, 1.11)	0.64
		CT/TT	56/127	0.8 (0.61, 1.05)	0.11
	***P_interaction_ serum GPx activity***				0.2

OR, 95% confidence interval (CI) and P values were calculated for each SNP analysed using logistic regression. OR adjusted for age group, family history of prostate cancer, participation in PSA testing, smoking status, and vigorous physical activity. P_interaction_ = P value of test for interaction between genotype and serum selenium concentration per 10 mg/l, serum SePP concentration (mg/l) or serum GPx3 activity per 100 U/l.

### Association of Genotype for SNPs in Selenoprotein Genes with Prostate Tumour Grade and Stage

Analysis of the data for the prostate cancer cases according to clinical parameters showed that within the study there were 69 advanced cases and 172 cases with localized disease, 90 individuals with high-grade tumours and 130 with low grade tumours. Regardless of markers of Se status, when tumour grade and disease stage were taken into account in the analysis there was a trend towards reduced risk of a high grade tumour in individuals carrying at least one T allele for rs28665122 in the gene *SEPS1* (OR = 0.57, 95% CI = 0–31–1.06, p = 0.08) (Table 3). Further analysis of the data incorporating both tumour category and markers of Se status was carried out. As shown in Table 4, when analysis was restricted to advanced stage prostate cancer, subjects homozygous for the C allele of rs7310505 in *TXNRD1* had an OR of 0.72 (95% CI = 0.56–0.93, p = 0.011) per 10 µg/l increase in serum Se concentration, whereas in subjects carrying the A allele (heterozygous and homozygous) no association was found (p_interaction_ = 0.02). Similarly, the C allele of rs7310505 was associated with lower risk of advanced cancer as serum SePP increased (OR 0.43 (95% CI = 0.21–0.88, p = 0.02). In contrast when analysis was restricted to local stage prostate cancer subjects there was no significant association of rs7310505 and either serum Se or SePP concentration with disease risk (Table 4). There was also evidence of lower risk of advanced prostate cancer in homozygous CC carriers for rs9605030 in the *TXNRD*2 gene (OR = 0.53, 95% CI = 0.29–0.95, p = 0.03) as serum SePP increased. No comparable differences were seen in local stage cancer patients. Restriction of analysis to subjects with high grade tumours showed that subjects carrying the T allele for rs28665122 (heterozygous and homozygous) in the *SEPS1* gene (OR = 0.46, 95% CI = 0.24–0.89, p = 0.02) were at lower risk of a high grade tumour per 10 µg/l increase in serum Se concentration but no association was observed in homozygous subjects where p_interaction_ did not reach statistical significance (Table 4).

**Table 3:** Odds ratio and 95% confidence interval for association of polymorphisms in selected selenoproteins with prostate cancer risk in strata of disease stage.

				Advanced cases		Localized disease		High grade		Low grade	
Gene		RS number	Genotype	OR(95%CI)	p-value	OR(95%CI)	p-value	OR(95%CI)	p-value	OR(95%CI)	p-value
*SELK*	rs9880056		TT	1		1		1		1	
			TC/CC	0.96 (0.54, 1.69)	0.89	1.26 (0.87, 1.84)	0.22	0.92 (0.55, 1.52)	0.73	1.28 (0.84, 1.95)	0.25
*TTXNRD2_1*	rs9605030		CC	1		1		1		1	
			CT/TT	1.4 (0.73, 2.69)	0.31	0.98 (0.64, 1.49)	0.91	1.19 (0.68, 2.08)	0.53	0.88 (0.53, 1.45)	0.61
*TXNRD2_2*	rs9605031		CC	1		1		1		1	
			CT/TT	1.09 (0.61, 1.95)	0.77	1.12 (0.78, 1.59)	0.54	0.97 (0.59, 1.58)	0.90	1.09 (0.71, 1.66)	0.69
*SBP2*	rs3211684		TT	1		1		1		1	
			GT/GG	1.07 (0.44, 2.64)	0.88	1.09 (0.64, 1.87)	0.7455	1.21 (0.52, 2.84)	0.66	0.92 (0.50, 1.70)	0.80
*TXNRD1*	rs7310505		CC	1		1		1		1	
			CA/AA	1.13 (0.63, 2.03)	0.69	1.13 (0.78, 1.63)	0.53	1.18 (0.71, 1.93)	0.52	0.9 (0.59, 1.39)	0.64
*SEPS1*	rs28665122		CC	1		1		1		1	
			CT/TT	0.93 (0.50, 1.76)	0.83	0.83 (0.54, 1.27)	0.39	0.57 (0.31, 1.06)	0.08	1.05 (0.66, 1.68)	0.84

OR, 95% confidence interval (CI) and P values were calculated for each SNP analysed using logistic regression and stratified according to disease stage. For each SNP, ORs are presented with reference to the most frequent homozygous genotype.

**Table 4:** Odds ratio and 95% confidence interval for association of markers of Se status and polymorphisms in selenoprotein genes with advanced and high-grade prostate c cancer in the EPIC-Heidelberg nested case-control study.

			Advanced stage		Localised disease		High grade			Low grade	
Gene	RS number	Genotype	OR (95%CI)	p-value	OR (95%CI)	p-value		OR (95%CI)	p-value	OR (95%CI)	p-value
*SELK*	rs9880056										
	rs9880056	TT	0.94 (0.72, 1.23)	0.64	0.93 (0.78, 1.10)	0.39		1 (0.80, 1.24)	1.00	0.9 (0.75, 1.10)	0.31
	rs9880056	TC/CC	0.67 (0.50, 0.89)	***0.01***	0.97 (0.81, 1.16)	0.76		0.76 (0.61, 0.94)	***0.01***	1.03 (0.82, 1.29)	0.80
	***P_interaction_ serum Se***			0.09		0.91			***0.05***		0.42
*SELK*	rs9880056										
	rs9880056	TT	0.78 (0.38, 1.62)	0.51	0.87 (0.55, 1.37)	0.55		1.2 (0.68, 2.11)	0.53	0.65 (0.38, 1.10)	0.11
	rs9880056	TC/CC	0.39 (0.16, 0.91)	***0.03***	0.95 (0.61, 1.46)	0.80		0.47 (0.26, 0.87)	***0.02***	1.11 (0.67, 1.85)	0.69
	***P_interaction_ serum SePP***			0.16		0.71			***0.01***		0.07
*SELK*	rs9880056										
	rs9880056	TT	0.78 (0.52, 1.16)	0.22	0.96 (0.80, 1.15)	0.64		1.02 (0.73, 1.43)	0.91	0.84 (0.67, 1.06)	0.14
	rs9880056	TC/CC	0.85 (0.59, 1.21)	0.36	0.94 (0.78, 1.13)	0.48		0.79 (0.61, 1.02)	0.08	0.98 (0.75, 1.27)	0.87
	***P_interaction_ serum GPx activity***			0.42		0.93			0.13		0.33
*TXNRD2_1*	rs9605030										
	rs9605030	CC	0.83 (0.68, 1.03)	0.09	1 (0.87, 1.14)	0.95		0.92 (0.77, 1.10)	0.37	0.97 (0.83, 1.13)	0.69
	rs9605030	CT/TT	0.77 (0.41, 1.44)	0.41	0.9 (0.68, 1.18)	0.43		0.7 (0.45, 1.08)	0.10	1.08 (0.74, 1.57)	0.68
	***P_interaction_ serum Se***			0.76		0.22			***0.05***		0.49
*TXNRD2_1*	rs9605030										
	rs9605030	CC	0.53 (0.29, 0.95)	***0.03***	0.98 (0.69, 1.40)	0.93		0.74 (0.47, 1.18)	0.21	0.92 (0.62, 1.37)	0.69
	rs9605030	CT/TT	0.56 (0.09, 3.66)	0.55	1.01 (0.52, 1.95)	0.99		0.95 (0.32, 2.76)	0.92	0.8 (0.31, 2.08)	0.65
	***P_interaction_ serum SePP***			0.44		0.86			0.74		0.84
*TXNRD2_1*	rs9605030										
	rs9605030	CC	0.76 (0.54, 1.07)	0.11	0.94 (0.81, 1.09)	0.43		0.86 (0.67, 1.10)	0.23	0.89 (0.74, 1.08)	0.24
	rs9605030	CT/TT	0.85 (0.47, 1.55)	0.60	0.98 (0.76, 1.27)	0.89		0.94 (0.61, 1.44)	0.77	1.08 (0.77, 1.50)	0.66
	***P_interaction_ serum GPx activity***			0.29		0.81			0.54		0.27
*TXNRD2_2*	rs9605031										
	rs9605031	CC	0.87 (0.68, 1.10)	0.24	1.03 (0.89, 1.19)	0.70		0.94 (0.78, 1.14)	0.53	1 (0.84, 1.20)	0.96
	rs9605031	CT/TT	0.72 (0.50, 1.04)	0.08	0.87 (0.71, 1.06)	0.17		0.73 (0.55, 0.97)	***0.03***	0.98 (0.76, 1.26)	0.85
	***P_interaction_ serum Se***			0.68		***0.02***			***0.02***		0.63
*TXNRD2_2*	rs9605031										
	rs9605031	CC	0.63 (0.32, 1.22)	0.17	0.97 (0.65, 1.44)	0.89		0.7 (0.42, 1.17)	0.17	0.98 (0.63, 1.53)	0.93
	rs9605031	CT/TT	0.44 (0.15, 1.27)	0.13	0.96 (0.58, 1.58)	0.87		0.77 (0.38, 1.56)	0.46	0.85 (0.45, 1.60)	0.62
	***P_interaction_ serum SePP***			0.71		0.85			0.71		0.83
*TXNRD2_2*	rs9605031										
	rs9605031	CC	0.86 (0.58, 1.26)	0.44	0.95 (0.79, 1.14)	0.60		0.88 (0.67, 1.16)	0.38	0.9 (0.72, 1.12)	0.35
	rs9605031	CT/TT	0.83 (0.54, 1.26)	0.38	0.96 (0.79, 1.17)	0.72		0.89 (0.66, 1.20)	0.45	0.97 (0.76, 1.23)	0.78
	***P_interaction_ serum GPx activity***			0.79		0.82			0.66		0.71
*SBP2*	rs3211684										
*SBP2*	rs3211684	GT/GG	– –	–	1.01 (0.63, 1.62)	0.96		– –	–	0.97 (0.84, 1.12)	0.66
*SBP2*	rs3211684	TT	0.84 (0.69, 1.02)	0.08	0.96 (0.85, 1.08)	0.50		0.89 (0.76, 1.04)	0.13	1.33 (0.67, 2.64)	0.42
	***P_interaction_ serum Se***			0.54		0.4			0.84		0.48
*SBP2*	rs3211684										
*SBP2*	rs3211684	GT/GG	– –	–	1.18 (0.33, 4.21)	0.80		0.72 (0.04, 13.53)	0.83	0.91 (0.62, 1.33)	0.62
*SBP2*	rs3211684	TT	0.6 (0.34, 1.04)	0.07	0.94 (0.68, 1.29)	0.70		0.75 (0.49, 1.14)	0.18	0.45 (0.06, 3.39)	0.44
	***P_interaction_ serum SePP***			0.81		0.48			0.4		0.96
*SBP2*	rs3211684										
*SBP2*	rs3211684	GT/GG	– –	–	0.96 (0.58, 1.60)	0.89		0.49 (0.13, 1.85)	0.29	0.92 (0.78, 1.09)	0.34
*SBP2*	rs3211684	TT	0.85 (0.64, 1.12)	0.24	0.95 (0.83, 1.08)	0.43		0.94 (0.77, 1.14)	0.51	1.02 (0.50, 2.05)	0.97
	***P_interaction_ serum GPx activity***			0.92		0.42			0.06		0.23
*TXNRD1*	rs7310505										
	rs7310505	CC	0.72 (0.56, 0.93)	***0.01***	0.96 (0.83, 1.11)	0.58		0.83 (0.68, 1.02)	0.07	0.95 (0.80, 1.13)	0.57
	rs7310505	CA/AA	0.99 (0.70, 1.40)	0.95	1.02 (0.83, 1.25)	0.83		0.95 (0.73, 1.23)	0.71	1.01 (0.79, 1.30)	0.91
	***P_interaction_ serum Se***			***0.02***		0.54			0.28		0.41
*TXNRD1*	rs7310505										
	rs7310505	CC	0.43 (0.21, 0.88)	***0.02***	1.05 (0.72, 1.52)	0.80		0.74 (0.44, 1.24)	0.25	0.92 (0.61, 1.40)	0.71
	rs7310505	CA/AA	1.2 (0.41, 3.53)	0.74	0.83 (0.47, 1.47)	0.51		0.68 (0.32, 1.44)	0.31	0.98 (0.45, 2.11)	0.95
	***P_interaction_ serum SePP***			***0.02***		0.37			0.73		1.00
*TXNRD1*	rs7310505										
	rs7310505	CC	0.88 (0.64, 1.22)	0.45	0.9 (0.75, 1.08)	0.25		0.96 (0.73, 1.26)	0.76	0.84 (0.68, 1.04)	0.11
	rs7310505	CA/AA	0.78 (0.47, 1.29)	0.34	1.03 (0.84, 1.26)	0.77		0.83 (0.60, 1.15)	0.26	1.11 (0.81, 1.52)	0.51
	***P_interaction_ serum GPx activity***			0.93		0.18			0.36		***0.02***
*SEPS1*	rs28665122										
*SEPS1*	rs28665122	CC	0.86 (0.70, 1.05)	0.14	1.01 (0.89, 1.15)	0.90		0.9 (0.76, 1.06)	0.22	1 (0.86, 1.18)	0.97
*SEPS1*	rs28665122	CT/TT	1.09 (0.53, 2.24)	0.81	0.88 (0.68, 1.16)	0.37		0.76 (0.50, 1.17)	0.21	0.89 (0.65, 1.22)	0.48
	***P_interaction_ serum Se***			0.48		0.08			0.16		0.22
*SEPS1*	rs28665122										
*SEPS1*	rs28665122	CC	0.62 (0.34, 1.11)	0.11	1.04 (0.73, 1.48)	0.81		0.8 (0.51, 1.26)	0.34	0.92 (0.61, 1.38)	0.69
*SEPS1*	rs28665122	CT/TT	1.24 (0.22, 7.17)	0.81	0.85 (0.43, 1.66)	0.63		0.46 (0.18, 1.21)	0.12	0.91 (0.40, 2.08)	0.82
	***P_interaction_ serum SePP***			0.94		0.14			0.32		0.78
*SEPS1*	rs28665122										
*SEPS1*	rs28665122	CC	0.91 (0.67, 1.22)	0.52	0.99 (0.85, 1.15)	0.89		0.95 (0.75, 1.20)	0.68	0.91 (0.75, 1.10)	0.33
*SEPS1*	rs28665122	CT/TT	0.58 (0.26, 1.30)	0.19	0.88 (0.63, 1.23)	0.45		0.46 (0.24, 0.89)	**0.02**	0.98 (0.67, 1.44)	0.92
	***P_interaction_ serum GPx activity***			0.12		0.2			0.22		0.94

Logistic regression adjusted for matching factors (age and time of blood collection) and family history of prostate cancer, participation in PSA testing, smoking status, and vigorous physical activity. Data was stratified according to disease stage. P_interaction_ = P value of test for interaction between genotype and serum selenium concentration per 10 mg/l, serum SePP concentration (mg/l) or serum GPx3 activity per 100 U/l.

Analysis of the data taking both clinical parameters and measures of Se status into account showed consistent association of rs9880056 in the *SELK* gene with advanced stage or high grade disease risk. As shown in Table 4, when analysis was restricted to advanced stage prostate cancer subjects with the C allele (homozygous and heterozygous) for rs9880056 had an OR of 0.67 (95% CI = 0.50–0.89, p = 0.006) per 10 µg/l increase in serum Se concentration. Similarly, the C allele of rs9880056 was associated with lower risk of advanced stage cancer as serum SePP increased (OR 0.39 (95% CI = 0.16–0.91, p = 0.029). When analysis was restricted to high grade prostate cancer, subjects with the C allele (homozygous and heterozygous) for rs9880056 had an OR of 0.76 (95% CI = 0.61–0.94, p = 0.01) per 10 µg/l increase in serum Se concentration (Table 4), whereas in subjects homozygous for the T allele no association was found (p_interaction_ = 0.05). Similarly, the C allele of rs9880056 was associated with lower risk of high grade tumour as serum SePP increased (OR 0.47 (95% CI = 0.26–0.87, p = 0.016)) with a p_interaction_ of 0.01. In addition there was a trend towards carriers of the C allele having a lower risk of advanced stage disease as serum GPx3 activity increased (OR = 0.79, 95% CI = 0.61–1.02, p = 0.08). Even when markers of Se status were included in the analysis no association of rs9880056 in *SELK* was found with risk of low grade tumour or local stage disease.

## Discussion

Although previous work has suggested a possible inverse association between Se levels and risk of prostate cancer [4], studies of the influence of genetic variants in selenoprotein genes on prostate cancer risk or survival have been limited [13], [17], [18]. These earlier studies have provided evidence that variants in a combination of the *SEPP1* and manganese superoxide dismutase (*SOD2*) genes affects disease risk [18], that rs1050450 in *GPX1* affects the influence of Se status on risk [13] and that variants in *SEP15* affect prostate cancer survival [17]. Increasingly, it is being realised that it is important to take an overall biological pathway approach to identify SNPs that show an association with a disease/disorder (e.g. [23], [24]). The present study used a two-phase genotyping approach to assess the influence of interaction of Se status and SNPs in genes across the selenium biological pathway on prostate cancer risk. The study extended earlier work by showing consistent significant interactions between serum markers of Se status and *TXNRD1*, *TXNRD2* and *SELK* genotype with respect to risk of high-grade or advanced stage prostate cancer. As we used tagging-SNPs it is difficult to evaluate the functionality of these variants. Both *TXNRD*2 variants and the *TXNRD1* variant studied are intronic and therefore the functional basis for the observed effects is unclear at present. In contrast, the SNP in *SELK* is in the promoter region and thus may influence gene expression through affecting promoter activity. A more detailed analysis of the genetic regions, combined with functional assays, could help in understanding the consequences of these SNPS or variants they are tagging.

The study involved analysis of samples from the EPIC-Heidelberg study and benefited from the availability of dietary and lifestyle data. However, the relatively small number of participants, particularly in the first phase of analysis, meant that the study was underpowered. Together with the lack of correction for multiple testing, this is a limitation that could lead to potential false positives that could have occurred by chance. However the strategy chosen here provides the opportunity to potentially identify new candidate functional SNPs and highlights the potential role of several selenoproteins in prostate function or prostate cancer etiology. Additionally, this approach reinforces previous observations [17] that the effects of some SNPs cannot be seen when Se status markers are not taken into account.

TR1 and TR2 proteins have major roles in regulation of redox signalling, whilst SelK has recently been reported to be an endoplasmic reticulum protein that is thought to play a role in the unfolded protein response and endoplasmic reticulum homeostasis [26], [27]. Both these biochemical processes are important in cell responses to metabolic challenges and are thought to be important in the control of cell proliferation and apoptosis. Therefore the observed effects of these SNPs are likely to reflect alterations in the ability of the prostate cells to respond to redox or inflammatory challenges. Interestingly, TR1 has been identified as one of four genes differentially expressed between androgen–dependent and independent growth of prostate cancer in mice [28]. Over-activation or dysfunction of thioredoxin reductases have been proposed to be involved in cancer etiology and prostate tumour progression [29], [30], [31]. As a result, thioredoxin reductases have been identified as anti-cancer targets and small molecules inhibitors of thioredoxin reductases (such as organoselenium compounds) are currently used in the treatment of prostate cancer [30], [31]. The observation that SNPs in both *TXNRD1* and *TXNRD2* genes are affecting prostate cancer risk supports the proposed role of the corresponding proteins in prostate function and/or tumour development. It would be interesting to determine if genetic variations in these two genes affect individual response to chemopreventive agents such as thioredoxin reductase inhibitors. In addition, SelK has been proposed to play a role in the regulation of Ca^2+^ flux [32] and changes in Ca^2+^ flux have been suggested to be involved in the progression to hormone-insensitive prostate cancer [33].

The association of SNPs in *TXNRD1* and *TXNRD2* with disease risk was only observed in advanced stage or high grade cancers and not in localized low-grade cases. This may reflect a role for these selenoproteins in the progression of prostate cancers rather that initiation, especially in view of the reported relationship between thioredoxin reductase activity and tumour aggressiveness [29]; alternatively, there may be different etiologies for the localized, low grade and the advanced, high grade cancers with different roles for the selenoproteins in the two disease situations. We hypothesize that sub-optimal Se status and altered activity of TR1, TR2 and SelK modify the ability of prostate cells to combat oxidative and inflammatory challenges and so affect their growth and tumour progression.

Selenoprotein expression in response to Se supplementation has been found to vary between individuals and some of these effects have been attributed to genetic polymorphisms in selenoproteins [34], [35], [36]. Synthesis of different selenoproteins responds differentially to sub-optimal Se intake [15], [37], [38] and since the protective effects of Se are thought to occur through selenoprotein biosynthesis one would expect potential genetic effects on prostate cancer risk to be modulated by Se status and *vice versa*. Since Se content of most foods depends on their geographical source Se intake is difficult to assess [1], but Se status can be assessed by measurement of blood biomarkers such as serum Se, plasma GPx or plasma SePP. SePP has been reported to be a better marker of status over a wider range of intake [12] than GPx3 but a recent systematic review concluded that more information is needed to evaluate their strengths and weaknesses as biomarkers of Se status [11]. Therefore, in this study we measured three markers of Se status and analysed them independently. There were significant interactions between Se status and *TXNRD1*, *TXNRD2* and *SELK* genotype with respect to high-grade or advanced stage prostate cancer, so emphasising the importance of the combination of Se intake and genotype in determining prostate cancer risk. Our observations that effects of Se-related SNPs on prostate cancer risk were observed only in combination with Se status provide a likely explanation of why gene variants in selenoprotein genes have not been identified as risk factors in a recent genome-wide association study [25].

The lack of significant association of genotype for single SNPs in *SEPP1* or *SEP15* on prostate cancer risk is consistent with earlier reports that also found no association of specific SNPs in these genes with prostate cancer risk [17], [18]. However, in a nested case control study [17] variants in *SEP15* were observed to be associated with prostate cancer mortality. In the present study we were unable to assess effects on mortality.

Large-scale Se supplementation trials in the USA have given contradictory outcomes with regards to evidence for a relationship between lower Se intake and increased risk of prostate cancer [5], [6]. This has been attributed to various factors, including higher baseline Se status in the SELECT study compared with the Nutritional Prevention of Cancer Trial [6]. The present study was carried out on European subjects in whom, on the basis of comparison of earlier populations [2], the mean Se status would be expected to be lower than in a US population. Indeed, as reported earlier [13] the mean plasma Se in control subjects within this study (87.7 µg/l) was lower than the baseline plasma Se in the recent SELECT trial [6]. In addition, there is evidence for familial associations of prostate cancer [39] and so it is also possible that the association between Se status and prostate cancer disease development may be modified by genetic variation in selenoproteins. Such genetic effects have been suggested to contribute to the differences in the outcomes of the Se supplementation trials [40].

In conclusion, this study shows a significant interaction between serum markers of Se status and *TXNRD1*, *TXNRD2* and *SELK* genotype with respect to high-grade or advanced stage prostate cancer. This complements a study of the same cohort that focused on a small number of functional selenoprotein SNPs [13]. The earlier data showed that genotype for rs1050450 in *GPX1* modified association of serum Se concentration with prostate cancer risk [13]. It also indicated that there was an association of borderline statistical between genotype for rs7579 in *SEPP1* and prostate cancer risk [13]. Thus overall, the data from this EPIC-Heidelberg nested case-control study indicate that together Se status and *GPX1*, *SEPP1*, *TXNRD1*, *TXNRD2,* and *SELK* genotype significantly alter risk of high-grade or advanced stage prostate cancer in a population with suboptimal Se intake. Future studies should not only address functional effects of these variants in prostate tissue and but also focus on the larger studies needed to investigate the complex interplay of polymorphisms in different selenoproteins and Se status in prostate cancer development. This work also illustrates that approaches that take multiple SNPs within a metabolic pathway into account are particularly relevant to the study of SNP-nutrient interactions in relation to the risk for a complex disease as they take into consideration the different components of a biological pathway and nutritional biomarkers; indeed pathway enrichment methods to analyse data from genome-wide association studies have been developed [24].

## Supporting Information

Table S1 Pathway-wise genotyping for SNPs in selenoprotein and related genes in control and prostate cancer patients from the EPIC-Heidelberg cohort. A custom chip was designed for genotyping across the whole pathway; the SNPs analysed and the corresponding genes are shown in the two left columns. Genotyping was carried out on 94 advanced cases and 94 control. Statistical evaluation of main effects of genetic variants on prostate cancer risk was carried out using either co-dominant and dominant models and data stratified for case set.
(DOC)
