# Supplementary material for: Polymorphisms in Thioredoxin Reductase and Selenoprotein K Genes and Selenium Status Modulate Risk of Prostate Cancer
Source: PLoS One. 2012 Nov 1;7(11):e48709. doi: 10.1371/journal.pone.0048709 (PMC3486803; doi:10.1371/journal.pone.0048709)
Supplement: Table S1 — Pathway-wise genotyping for SNPs in selenoprotein and related genes in control and prostate cancer patients from the EPIC-Heidelberg cohort. A custom chip was designed for genotyping across the whole pathway; the SNPs analysed and the corresponding genes are shown in the two left columns. Genotyping was carried out on 94 advanced cases and 94 control. Statistical evaluation of main effects of genetic variants on prostate cancer risk was carried out using either co-dominant and dominant models and data stratified for case set. (DOC) [file pone.0048709.s001.doc]

**Supplementary Table 1. Pathway-wise genotyping for SNPs in selenoprotein and related genes in control and prostate cancer patients from the EPIC-Heidelberg cohort.** A custom chip was designed for genotyping across the whole pathway; the SNPs analysed and the corresponding genes are shown in the two left columns.Genotyping was carried out on 94 advanced cases and 94 control. Statistical evaluation of main effects of genetic variants on prostate cancer risk was carried out using either co-dominant and dominant models and data stratified for case set.

| Gene | SNP | Allele | Cases / Controls | Wild vs. Heterozygot vs. Mutant | | | Wild vs. Others | | Significant (p< 0.1) |
| --- | --- | --- | --- | --- | --- | --- | --- | --- | --- |
|  |  |  |  | OR (CI) | p-Value | p-Trend | OR (CI) | p-Trend |  |
| -99 | rs5748469 | C C | 43 / 41 | 1.00 |  | 0.920 | 1.00 | 0.763 |  |
|  |  | C A | 33 / 38 | 0.80 (0.41; 1.57) | 0.521 |  | 0.91 (0.51; 1.65) |  |  |
|  |  | A A | 15 / 12 | 1.17 (0.51; 2.69) | 0.716 |  |  |  |  |
| ACTR8 | rs13073855 | C C | 82 / 75 | 1.00 |  | 0.151 | 1.00 | 0.151 |  |
|  |  | C G | 9 / 16 | 0.53 (0.23; 1.26) | 0.151 |  | 0.53 (0.23; 1.26) |  |  |
| ALOX5 | rs10751382 | A A | 31 / 40 | 1.00 |  | 0.377 | 1.00 | 0.210 |  |
|  |  | A G | 45 / 36 | 1.51 (0.83; 2.74) | 0.180 |  | 1.43 (0.82; 2.50) |  |  |
|  |  | G G | 15 / 15 | 1.23 (0.54; 2.81) | 0.628 |  |  |  |  |
| ALOX5 | rs10900213 | A A | 22 / 34 | 1.00 |  | 0.053 | 1.00 | 0.062 | * |
|  |  | A C | 48 / 43 | 1.70 (0.84; 3.42) | 0.139 |  | 1.86 (0.97; 3.56) |  |  |
|  |  | C C | 21 / 14 | 2.26 (0.94; 5.43) | 0.069 |  |  |  | * |
| ALOX5 | rs11239515 | A A | 71 / 63 | 1.00 |  | 0.179 | 1.00 | 0.186 |  |
|  |  | A G | 17 / 23 | 0.67 (0.32; 1.38) | 0.277 |  | 0.64 (0.33; 1.24) |  |  |
|  |  | G G | 2 / 4 | 0.50 (0.09; 2.73) | 0.424 |  |  |  |  |
| ALOX5 | rs1487562 | G G | 57 / 60 | 1.00 |  | 1.000 | 1.00 | 0.655 |  |
|  |  | G A | 28 / 22 | 1.30 (0.69; 2.46) | 0.415 |  | 1.14 (0.64; 2.05) |  |  |
|  |  | A A | 5 / 8 | 0.68 (0.22; 2.11) | 0.500 |  |  |  |  |
| ALOX5 | rs1565096 | A A | 38 / 53 | 1.00 |  | 0.057 | 1.00 | 0.035 | * |
|  |  | A G | 45 / 32 | 1.89 (1.03; 3.47) | 0.041 |  | 1.88 (1.05; 3.39) |  | * |
|  |  | G G | 8 / 6 | 1.85 (0.60; 5.65) | 0.283 |  |  |  |  |
| ALOX5 | rs2115819 | A A | 27 / 23 | 1.00 |  | 0.487 | 1.00 | 0.528 |  |
|  |  | A G | 46 / 47 | 0.85 (0.43; 1.67) | 0.640 |  | 0.82 (0.44; 1.53) |  |  |
|  |  | G G | 18 / 21 | 0.76 (0.35; 1.67) | 0.494 |  |  |  |  |
| ALOX5 | rs2279435 | A A | 58 / 67 | 1.00 |  | 0.088 | 1.00 | 0.058 | * |
|  |  | A C | 4 / 1 | 4.82 (0.52; 44.89) | 0.167 |  | 2.50 (0.97; 6.44) |  |  |
|  |  | C C | 15 / 9 | 2.20 (0.81; 6.01) | 0.124 |  |  |  |  |
| ALOX5 | rs3780894 | A A | 71 / 71 | 1.00 |  | 0.790 | 1.00 | 1.000 |  |
|  |  | A G | 17 / 15 | 1.13 (0.54; 2.39) | 0.740 |  | 1.00 (0.51; 1.96) |  |  |
|  |  | G G | 3 / 5 | 0.61 (0.15; 2.56) | 0.499 |  |  |  |  |
| ALOX5 | rs3780914 | G G | 32 / 29 | 1.00 |  | 0.347 | 1.00 | 0.640 |  |
|  |  | G A | 46 / 42 | 1.07 (0.52; 2.20) | 0.850 |  | 0.86 (0.47; 1.60) |  |  |
|  |  | A A | 13 / 20 | 0.67 (0.31; 1.43) | 0.301 |  |  |  |  |
| ALOX5 | rs3824612 | G G | 40 / 29 | 1.00 |  | 0.068 | 1.00 | 0.090 | * |
|  |  | G A | 36 / 40 | 0.64 (0.32; 1.26) | 0.196 |  | 0.58 (0.31; 1.09) |  |  |
|  |  | A A | 15 / 22 | 0.46 (0.19; 1.10) | 0.083 |  |  |  | * |
| ALOX5 | rs4948672 | G G | 78 / 68 | 1.00 |  | 0.082 | 1.00 | 0.082 | * |
|  |  | G C | 13 / 23 | 0.52 (0.25; 1.09) | 0.082 |  | 0.52 (0.25; 1.09) |  | * |
| ALOX5 | rs7099684 | T T | 45 / 60 | 1.00 |  | 0.030 | 1.00 | 0.028 | * |
|  |  | T A | 38 / 27 | 1.91 (0.99; 3.67) | 0.053 |  | 2.00 (1.08; 3.72) |  | * |
|  |  | A A | 8 / 4 | 2.52 (0.73; 8.68) | 0.144 |  |  |  |  |
| ALOX5 | rs745986 | A A | 61 / 57 | 1.00 |  | 0.367 | 1.00 | 0.572 |  |
|  |  | A G | 27 / 27 | 0.98 (0.54; 1.80) | 0.951 |  | 0.85 (0.49; 1.49) |  |  |
|  |  | G G | 3 / 7 | 0.43 (0.11; 1.66) | 0.219 |  |  |  |  |
| C9orf164 | rs4876978 | G G | 50 / 50 | 1.00 |  | 0.584 | 1.00 | 1.000 |  |
|  |  | G A | 30 / 35 | 0.82 (0.43; 1.56) | 0.551 |  | 1.00 (0.56; 1.78) |  |  |
|  |  | A A | 11 / 6 | 1.90 (0.64; 5.63) | 0.247 |  |  |  |  |
| CELSR2 | rs17035415 | C C | 55 / 59 | 1.00 |  | 0.793 | 1.00 | 0.556 |  |
|  |  | C A | 34 / 28 | 1.33 (0.72; 2.46) | 0.356 |  | 1.19 (0.67; 2.13) |  |  |
|  |  | A A | 1 / 3 | 0.33 (0.04; 3.21) | 0.341 |  |  |  |  |
| CHSY1 | rs12900538 | A A | 41 / 30 | 1.00 |  | 0.214 | 1.00 | 0.097 | * |
|  |  | A G | 35 / 45 | 0.55 (0.28; 1.09) | 0.085 |  | 0.59 (0.32; 1.10) |  | * |
|  |  | G G | 15 / 16 | 0.71 (0.29; 1.70) | 0.436 |  |  |  |  |
| CHSY1 | rs4965809 | C C | 58 / 51 | 1.00 |  | 0.364 | 1.00 | 0.288 |  |
|  |  | C G | 29 / 36 | 0.71 (0.38; 1.32) | 0.277 |  | 0.72 (0.39; 1.32) |  |  |
|  |  | G G | 4 / 4 | 0.84 (0.20; 3.50) | 0.813 |  |  |  |  |
| CHSY1 | rs4965810 | A A | 65 / 61 | 1.00 |  | 0.536 | 1.00 | 0.556 |  |
|  |  | A G | 23 / 26 | 0.86 (0.46; 1.59) | 0.623 |  | 0.84 (0.47; 1.50) |  |  |
|  |  | G G | 3 / 4 | 0.73 (0.16; 3.29) | 0.686 |  |  |  |  |
| CHURC1 | rs9323457 | G G | 78 / 78 | 1.00 |  | 0.842 | 1.00 | 1.000 |  |
|  |  | G C | 13 / 12 | 1.10 (0.47; 2.59) | 0.827 |  | 1.00 (0.43; 2.31) |  |  |
|  |  | C C | 0 / 1 | 0.00 (0.00; .) | 0.994 |  |  |  |  |
| CKS2 | rs11137559 | G G | 40 / 40 | 1.00 |  | 0.800 | 1.00 | 1.000 |  |
|  |  | G A | 42 / 44 | 0.96 (0.53; 1.76) | 0.902 |  | 1.00 (0.55; 1.81) |  |  |
|  |  | A A | 9 / 7 | 1.47 (0.39; 5.47) | 0.569 |  |  |  |  |
| CKS2 | rs12236534 | A A | 70 / 75 | 1.00 |  | 0.278 | 1.00 | 0.400 |  |
|  |  | A G | 19 / 16 | 1.20 (0.61; 2.38) | 0.602 |  | 1.33 (0.68; 2.60) |  |  |
|  |  | G G | 2 / 0 | 4004360.21 (0.00; .) | 0.991 |  |  |  |  |
| CKS2 | rs13293841 | T T | 38 / 34 | 1.00 |  | 0.829 | 1.00 | 0.564 |  |
|  |  | T A | 40 / 50 | 0.70 (0.38; 1.30) | 0.260 |  | 0.85 (0.48; 1.49) |  |  |
|  |  | A A | 13 / 7 | 1.72 (0.62; 4.74) | 0.296 |  |  |  |  |
| CKS2 | rs13294002 | A A | 65 / 63 | 1.00 |  | 0.773 | 1.00 | 0.739 |  |
|  |  | A C | 21 / 27 | 0.79 (0.40; 1.55) | 0.493 |  | 0.90 (0.47; 1.72) |  |  |
|  |  | C C | 5 / 1 | 4.46 (0.51; 39.13) | 0.177 |  |  |  |  |
| CKS2 | rs1556046 | A A | 32 / 30 | 1.00 |  | 0.920 | 1.00 | 0.763 |  |
|  |  | A C | 42 / 45 | 0.87 (0.46; 1.67) | 0.678 |  | 0.91 (0.51; 1.65) |  |  |
|  |  | C C | 17 / 16 | 1.01 (0.45; 2.28) | 0.985 |  |  |  |  |
| CKS2 | rs3211673 | A A | 82 / 79 | 1.00 |  | 0.352 | 1.00 | 0.442 |  |
|  |  | A C | 9 / 11 | 0.75 (0.26; 2.16) | 0.594 |  | 0.67 (0.24; 1.87) |  |  |
|  |  | C C | 0 / 1 | 0.00 (0.00; .) | 0.994 |  |  |  |  |
| CKS2 | rs3211679 | A A | 70 / 78 | 1.00 |  | 0.167 | 1.00 | 0.110 |  |
|  |  | A T | 20 / 11 | 2.00 (0.86; 4.67) | 0.109 |  | 2.00 (0.86; 4.67) |  |  |
|  |  | T T | 0 / 1 | 0.00 (0.00; .) | 0.994 |  |  |  |  |
| CKS2 | rs3211684 | A A | 82 / 84 | 1.00 |  | 0.471 | 1.00 | 0.594 |  |
|  |  | A C | 8 / 7 | 1.17 (0.39; 3.47) | 0.782 |  | 1.33 (0.46; 3.84) |  |  |
|  |  | C C | 1 / 0 | 4004360.21 (0.00; .) | 0.994 |  |  |  |  |
| CKS2 | rs3211698 | A A | 88 / 84 | 1.00 |  | 0.413 | 1.00 | 0.220 |  |
|  |  | A G | 2 / 7 | 0.29 (0.06; 1.38) | 0.118 |  | 0.43 (0.11; 1.66) |  |  |
|  |  | G G | 1 / 0 | 4004360.21 (0.00; .) | 0.994 |  |  |  |  |
| CKS2 | rs3211703 | A A | 65 / 70 | 1.00 |  | 0.330 | 1.00 | 0.425 |  |
|  |  | A G | 23 / 20 | 1.19 (0.61; 2.31) | 0.613 |  | 1.29 (0.69; 2.44) |  |  |
|  |  | G G | 3 / 1 | 3.00 (0.31; 28.84) | 0.341 |  |  |  |  |
| CLP1 | rs11229087 | G G | 43 / 43 | 1.00 |  | 0.523 | 1.00 | 1.000 |  |
|  |  | G A | 38 / 43 | 0.95 (0.51; 1.78) | 0.873 |  | 1.00 (0.54; 1.86) |  |  |
|  |  | A A | 10 / 5 | 1.92 (0.59; 6.27) | 0.280 |  |  |  |  |
| CTNND1 | rs11570210 | A A | 79 / 79 | 1.00 |  | 1.000 | 1.00 | 1.000 |  |
|  |  | A G | 12 / 12 | 1.00 (0.38; 2.66) | 1.000 |  | 1.00 (0.38; 2.66) |  |  |
| CTNND1 | rs11601320 | A A | 44 / 43 | 1.00 |  | 0.827 | 1.00 | 0.882 |  |
|  |  | A G | 38 / 38 | 0.98 (0.52; 1.82) | 0.940 |  | 0.96 (0.53; 1.72) |  |  |
|  |  | G G | 9 / 10 | 0.89 (0.35; 2.30) | 0.810 |  |  |  |  |
| CTNND1 | rs12790660 | A A | 47 / 43 | 1.00 |  | 0.748 | 1.00 | 0.556 |  |
|  |  | A G | 34 / 39 | 0.79 (0.42; 1.48) | 0.468 |  | 0.84 (0.47; 1.50) |  |  |
|  |  | G G | 10 / 9 | 1.01 (0.39; 2.58) | 0.987 |  |  |  |  |
| CTNND1 | rs2156638 | C C | 31 / 29 | 1.00 |  | 0.406 | 1.00 | 0.773 |  |
|  |  | C G | 49 / 45 | 1.00 (0.56; 1.81) | 0.991 |  | 0.92 (0.52; 1.62) |  |  |
|  |  | G G | 11 / 17 | 0.63 (0.26; 1.51) | 0.297 |  |  |  |  |
| CTNND1 | rs567017 | T T | 47 / 51 | 1.00 |  | 0.260 | 1.00 | 0.538 |  |
|  |  | T A | 35 / 36 | 1.13 (0.61; 2.10) | 0.699 |  | 1.21 (0.66; 2.22) |  |  |
|  |  | A A | 9 / 4 | 2.45 (0.70; 8.60) | 0.162 |  |  |  |  |
| CTNND1 | rs592785 | A A | 32 / 30 | 1.00 |  | 0.597 | 1.00 | 0.778 |  |
|  |  | A G | 49 / 48 | 0.96 (0.54; 1.70) | 0.889 |  | 0.92 (0.53; 1.61) |  |  |
|  |  | G G | 10 / 13 | 0.75 (0.30; 1.85) | 0.532 |  |  |  |  |
| DIO1 | rs11206244 | G G | 51 / 40 | 1.00 |  | 0.095 | 1.00 | 0.075 | * |
|  |  | G A | 31 / 39 | 0.55 (0.28; 1.12) | 0.098 |  | 0.54 (0.28; 1.06) |  | * |
|  |  | A A | 9 / 12 | 0.49 (0.17; 1.43) | 0.191 |  |  |  |  |
| DIO1 | rs12033572 | G G | 86 / 85 | 1.00 |  | 0.763 | 1.00 | 0.763 |  |
|  |  | G C | 5 / 6 | 0.83 (0.25; 2.73) | 0.763 |  | 0.83 (0.25; 2.73) |  |  |
| DIO1 | rs12131461 | G G | 63 / 68 | 1.00 |  | 0.501 | 1.00 | 0.425 |  |
|  |  | G A | 24 / 19 | 1.34 (0.68; 2.61) | 0.399 |  | 1.29 (0.69; 2.44) |  |  |
|  |  | A A | 3 / 3 | 1.05 (0.21; 5.23) | 0.953 |  |  |  |  |
| DIO1 | rs17109582 | A A | 71 / 75 | 1.00 |  | 0.347 | 1.00 | 0.451 |  |
|  |  | A G | 17 / 15 | 1.18 (0.53; 2.64) | 0.683 |  | 1.33 (0.63; 2.82) |  |  |
|  |  | G G | 3 / 1 | 3.00 (0.31; 28.84) | 0.341 |  |  |  |  |
| DIO1 | rs2235544 | A A | 26 / 28 | 1.00 |  | 0.225 | 1.00 | 0.739 |  |
|  |  | A C | 34 / 41 | 0.97 (0.49; 1.92) | 0.933 |  | 1.12 (0.58; 2.15) |  |  |
|  |  | C C | 31 / 22 | 1.86 (0.75; 4.62) | 0.181 |  |  |  |  |
| DIO1 | rs2268181 | A A | 62 / 66 | 1.00 |  | 0.448 | 1.00 | 0.538 |  |
|  |  | A G | 24 / 22 | 1.14 (0.60; 2.17) | 0.683 |  | 1.21 (0.66; 2.22) |  |  |
|  |  | G G | 5 / 3 | 1.72 (0.41; 7.29) | 0.459 |  |  |  |  |
| DIO1 | rs2284454 | A A | 56 / 64 | 1.00 |  | 0.305 | 1.00 | 0.220 |  |
|  |  | A G | 31 / 23 | 1.52 (0.80; 2.88) | 0.203 |  | 1.47 (0.79; 2.72) |  |  |
|  |  | G G | 4 / 4 | 1.17 (0.28; 4.80) | 0.829 |  |  |  |  |
| DIO1 | rs2294511 | T T | 46 / 35 | 1.00 |  | 0.148 | 1.00 | 0.105 |  |
|  |  | T A | 35 / 44 | 0.61 (0.33; 1.14) | 0.120 |  | 0.61 (0.33; 1.11) |  |  |
|  |  | A A | 10 / 12 | 0.60 (0.22; 1.65) | 0.325 |  |  |  |  |
| DIO1 | rs731828 | A A | 35 / 32 | 1.00 |  | 0.842 | 1.00 | 0.669 |  |
|  |  | A C | 36 / 44 | 0.74 (0.40; 1.38) | 0.342 |  | 0.89 (0.51; 1.55) |  |  |
|  |  | C C | 20 / 15 | 1.39 (0.58; 3.31) | 0.464 |  |  |  |  |
| DIO2 | rs12885300 | G G | 32 / 37 | 1.00 |  | 0.229 | 1.00 | 0.447 |  |
|  |  | G A | 38 / 40 | 1.11 (0.58; 2.12) | 0.759 |  | 1.26 (0.69; 2.31) |  |  |
|  |  | A A | 21 / 14 | 1.73 (0.75; 3.98) | 0.197 |  |  |  |  |
| DIO2 | rs1907526 | G G | 78 / 71 | 1.00 |  | 0.339 | 1.00 | 0.167 |  |
|  |  | G A | 11 / 20 | 0.56 (0.25; 1.27) | 0.167 |  | 0.56 (0.25; 1.27) |  |  |
|  |  | A A | 2 / 0 | 2252452.62 (0.00; .) | 0.992 |  |  |  |  |
| DIO2 | rs224995 | A A | 72 / 63 | 1.00 |  | 0.357 | 1.00 | 0.143 |  |
|  |  | A G | 16 / 28 | 0.57 (0.29; 1.12) | 0.100 |  | 0.61 (0.31; 1.18) |  |  |
|  |  | G G | 3 / 0 | 7604643.20 (0.00; .) | 0.993 |  |  |  |  |
| DIO2 | rs225011 | A A | 32 / 34 | 1.00 |  | 0.569 | 1.00 | 0.752 |  |
|  |  | A G | 43 / 44 | 1.05 (0.55; 2.00) | 0.888 |  | 1.11 (0.59; 2.06) |  |  |
|  |  | G G | 16 / 13 | 1.37 (0.53; 3.56) | 0.515 |  |  |  |  |
| DIO2 | rs225014 | A A | 40 / 44 | 1.00 |  | 0.320 | 1.00 | 0.556 |  |
|  |  | A G | 38 / 39 | 1.09 (0.60; 2.00) | 0.771 |  | 1.19 (0.67; 2.13) |  |  |
|  |  | G G | 13 / 8 | 1.81 (0.67; 4.89) | 0.245 |  |  |  |  |
| DIO2 | rs225015 | G G | 42 / 51 | 1.00 |  | 0.184 | 1.00 | 0.192 |  |
|  |  | G A | 39 / 33 | 1.42 (0.77; 2.63) | 0.266 |  | 1.47 (0.82; 2.64) |  |  |
|  |  | A A | 10 / 7 | 1.73 (0.60; 5.04) | 0.314 |  |  |  |  |
| DIO3 | rs945006 | A A | 67 / 65 | 1.00 |  | 0.876 | 1.00 | 0.746 |  |
|  |  | A C | 22 / 25 | 0.85 (0.45; 1.62) | 0.622 |  | 0.90 (0.48; 1.70) |  |  |
|  |  | C C | 1 / 0 | 4004360.21 (0.00; .) | 0.994 |  |  |  |  |
| EEFSEC | rs11718942 | A A | 72 / 75 | 1.00 |  | 0.602 | 1.00 | 0.602 |  |
|  |  | A C | 19 / 16 | 1.20 (0.61; 2.38) | 0.602 |  | 1.20 (0.61; 2.38) |  |  |
| EEFSEC | rs12632366 | G G | 27 / 17 | 1.00 |  | 0.739 | 1.00 | 0.091 | * |
|  |  | G A | 39 / 62 | 0.37 (0.17; 0.81) | 0.013 |  | 0.55 (0.27; 1.10) |  | * |
|  |  | A A | 25 / 12 | 1.39 (0.53; 3.61) | 0.505 |  |  |  |  |
| EEFSEC | rs1702134 | C C | 69 / 71 | 1.00 |  | 0.506 | 1.00 | 0.716 |  |
|  |  | C A | 20 / 20 | 1.00 (0.48; 2.10) | 1.000 |  | 1.14 (0.56; 2.34) |  |  |
|  |  | A A | 2 / 0 | 4004360.21 (0.00; .) | 0.991 |  |  |  |  |
| EEFSEC | rs1735546 | A A | 50 / 47 | 1.00 |  | 1.000 | 1.00 | 0.655 |  |
|  |  | A C | 35 / 41 | 0.82 (0.45; 1.49) | 0.509 |  | 0.88 (0.49; 1.57) |  |  |
|  |  | C C | 6 / 3 | 1.79 (0.43; 7.44) | 0.423 |  |  |  |  |
| EEFSEC | rs1735562 | G G | 77 / 63 | 1.00 |  | 0.042 | 1.00 | 0.023 | * |
|  |  | G A | 13 / 28 | 0.40 (0.19; 0.83) | 0.014 |  | 0.44 (0.22; 0.89) |  | * |
|  |  | A A | 1 / 0 | 4004360.21 (0.00; .) | 0.994 |  |  |  |  |
| EEFSEC | rs17401543 | G G | 82 / 72 | 1.00 |  | 0.056 | 1.00 | 0.056 | * |
|  |  | G A | 9 / 19 | 0.45 (0.19; 1.02) | 0.056 |  | 0.45 (0.19; 1.02) |  | * |
| EEFSEC | rs2293947 | A A | 74 / 77 | 1.00 |  | 0.591 | 1.00 | 0.591 |  |
|  |  | A C | 17 / 14 | 1.21 (0.60; 2.46) | 0.591 |  | 1.21 (0.60; 2.46) |  |  |
| EEFSEC | rs2999031 | A A | 27 / 24 | 1.00 |  | 0.667 | 1.00 | 0.622 |  |
|  |  | A T | 42 / 52 | 0.72 (0.36; 1.43) | 0.349 |  | 0.85 (0.45; 1.62) |  |  |
|  |  | T T | 22 / 15 | 1.32 (0.55; 3.16) | 0.528 |  |  |  |  |
| EEFSEC | rs2999064 | G G | 64 / 64 | 1.00 |  | 0.889 | 1.00 | 1.000 |  |
|  |  | G C | 25 / 26 | 0.95 (0.51; 1.78) | 0.873 |  | 1.00 (0.55; 1.83) |  |  |
|  |  | C C | 2 / 1 | 2.00 (0.18; 22.05) | 0.572 |  |  |  |  |
| EEFSEC | rs2999068 | G G | 72 / 69 | 1.00 |  | 0.746 | 1.00 | 0.613 |  |
|  |  | G A | 17 / 21 | 0.79 (0.40; 1.55) | 0.494 |  | 0.84 (0.43; 1.64) |  |  |
|  |  | A A | 1 / 0 | 4004360.21 (0.00; .) | 0.994 |  |  |  |  |
| EEFSEC | rs3122174 | A A | 64 / 64 | 1.00 |  | 0.889 | 1.00 | 1.000 |  |
|  |  | A C | 25 / 26 | 0.95 (0.51; 1.78) | 0.873 |  | 1.00 (0.55; 1.83) |  |  |
|  |  | C C | 2 / 1 | 2.00 (0.18; 22.05) | 0.572 |  |  |  |  |
| EEFSEC | rs4293718 | G G | 53 / 49 | 1.00 |  | 0.529 | 1.00 | 0.564 |  |
|  |  | G A | 33 / 36 | 0.86 (0.48; 1.52) | 0.593 |  | 0.85 (0.48; 1.49) |  |  |
|  |  | A A | 4 / 5 | 0.72 (0.18; 2.84) | 0.639 |  |  |  |  |
| EEFSEC | rs4857871 | A A | 48 / 44 | 1.00 |  | 0.901 | 1.00 | 0.556 |  |
|  |  | A G | 37 / 44 | 0.77 (0.42; 1.40) | 0.389 |  | 0.84 (0.47; 1.50) |  |  |
|  |  | G G | 6 / 3 | 1.78 (0.43; 7.31) | 0.422 |  |  |  |  |
| EEFSEC | rs729847 | G G | 53 / 56 | 1.00 |  | 0.356 | 1.00 | 0.648 |  |
|  |  | G A | 33 / 34 | 1.02 (0.55; 1.89) | 0.958 |  | 1.15 (0.63; 2.09) |  |  |
|  |  | A A | 5 / 1 | 5.03 (0.58; 43.47) | 0.142 |  |  |  |  |
| EEFSEC | rs7374952 | G G | 71 / 68 | 1.00 |  | 0.746 | 1.00 | 0.613 |  |
|  |  | G A | 17 / 21 | 0.79 (0.40; 1.55) | 0.494 |  | 0.84 (0.43; 1.64) |  |  |
|  |  | A A | 1 / 0 | 4004360.21 (0.00; .) | 0.994 |  |  |  |  |
| EEFSEC | rs9856329 | C C | 64 / 71 | 1.00 |  | 0.437 | 1.00 | 0.240 |  |
|  |  | C A | 27 / 18 | 1.75 (0.86; 3.56) | 0.122 |  | 1.50 (0.76; 2.95) |  |  |
|  |  | A A | 0 / 2 | 0.00 (0.00; .) | 0.991 |  |  |  |  |
| EID2B | rs17795475 | A A | 48 / 43 | 1.00 |  | 0.394 | 1.00 | 0.436 |  |
|  |  | A C | 37 / 40 | 0.81 (0.42; 1.55) | 0.528 |  | 0.78 (0.42; 1.45) |  |  |
|  |  | C C | 6 / 8 | 0.65 (0.20; 2.13) | 0.482 |  |  |  |  |
| EIF2A | rs2049229 | A A | 49 / 32 | 1.00 |  | 0.086 | 1.00 | 0.018 | * |
|  |  | A C | 29 / 46 | 0.38 (0.19; 0.78) | 0.008 |  | 0.49 (0.27; 0.88) |  | * |
|  |  | C C | 12 / 12 | 0.81 (0.31; 2.11) | 0.672 |  |  |  |  |
| EIF2A | rs2090916 | G G | 67 / 61 | 1.00 |  | 0.339 | 1.00 | 0.332 |  |
|  |  | G A | 21 / 26 | 0.74 (0.38; 1.43) | 0.365 |  | 0.73 (0.38; 1.39) |  |  |
|  |  | A A | 3 / 4 | 0.66 (0.14; 3.03) | 0.590 |  |  |  |  |
| ESPNL | rs10186514 | G G | 79 / 68 | 1.00 |  | 0.030 | 1.00 | 0.034 | * |
|  |  | G A | 12 / 22 | 0.41 (0.17; 0.99) | 0.048 |  | 0.39 (0.16; 0.93) |  | * |
|  |  | A A | 0 / 1 | 0.00 (0.00; .) | 0.994 |  |  |  |  |
| ESPNL | rs10210979 | G G | 61 / 64 | 1.00 |  | 1.000 | 1.00 | 0.578 |  |
|  |  | G A | 27 / 21 | 1.44 (0.66; 3.12) | 0.361 |  | 1.23 (0.59; 2.56) |  |  |
|  |  | A A | 3 / 6 | 0.46 (0.09; 2.47) | 0.368 |  |  |  |  |
| ESPNL | rs12692210 | G G | 41 / 45 | 1.00 |  | 0.819 | 1.00 | 0.572 |  |
|  |  | G A | 48 / 38 | 1.52 (0.82; 2.79) | 0.183 |  | 1.17 (0.67; 2.05) |  |  |
|  |  | A A | 2 / 8 | 0.27 (0.06; 1.28) | 0.099 |  |  |  | * |
| ESPNL | rs2121406 | G G | 24 / 31 | 1.00 |  | 0.123 | 1.00 | 0.198 |  |
|  |  | G A | 41 / 40 | 1.48 (0.67; 3.24) | 0.330 |  | 1.64 (0.77; 3.46) |  |  |
|  |  | A A | 24 / 18 | 2.17 (0.81; 5.85) | 0.125 |  |  |  |  |
| ESPNL | rs3948117 | A A | 58 / 61 | 1.00 |  | 0.769 | 1.00 | 0.622 |  |
|  |  | A G | 32 / 28 | 1.27 (0.64; 2.49) | 0.494 |  | 1.18 (0.62; 2.25) |  |  |
|  |  | G G | 1 / 2 | 0.50 (0.05; 5.51) | 0.572 |  |  |  |  |
| ESPNL | rs4663291 | G G | 65 / 68 | 1.00 |  | 0.778 | 1.00 | 0.640 |  |
|  |  | G A | 24 / 20 | 1.24 (0.65; 2.34) | 0.517 |  | 1.16 (0.63; 2.14) |  |  |
|  |  | A A | 1 / 2 | 0.50 (0.05; 5.51) | 0.572 |  |  |  |  |
| ESPNL | rs6728338 | G G | 84 / 82 | 1.00 |  | 0.594 | 1.00 | 0.594 |  |
|  |  | G A | 7 / 9 | 0.75 (0.26; 2.16) | 0.594 |  | 0.75 (0.26; 2.16) |  |  |
| ESPNL | rs7565244 | G G | 49 / 48 | 1.00 |  | 1.000 | 1.00 | 0.876 |  |
|  |  | G A | 33 / 35 | 0.92 (0.48; 1.76) | 0.801 |  | 0.95 (0.52; 1.76) |  |  |
|  |  | A A | 9 / 8 | 1.10 (0.38; 3.17) | 0.861 |  |  |  |  |
| ESPNL | rs7583402 | A A | 49 / 44 | 1.00 |  | 0.215 | 1.00 | 0.436 |  |
|  |  | A G | 38 / 38 | 0.89 (0.47; 1.69) | 0.710 |  | 0.78 (0.42; 1.45) |  |  |
|  |  | G G | 4 / 9 | 0.35 (0.09; 1.38) | 0.135 |  |  |  |  |
| ESPNL | rs878133 | G G | 67 / 66 | 1.00 |  | 1.000 | 1.00 | 0.870 |  |
|  |  | G A | 23 / 25 | 0.90 (0.47; 1.72) | 0.739 |  | 0.95 (0.50; 1.81) |  |  |
|  |  | A A | 1 / 0 | 4004360.21 (0.00; .) | 0.994 |  |  |  |  |
| FAM54B | rs11247764 | C C | 22 / 24 | 1.00 |  | 0.593 | 1.00 | 0.732 |  |
|  |  | C G | 46 / 47 | 1.04 (0.49; 2.19) | 0.916 |  | 1.13 (0.57; 2.21) |  |  |
|  |  | G G | 23 / 20 | 1.28 (0.54; 2.99) | 0.575 |  |  |  |  |
| FOS | rs1063169 | C C | 73 / 59 | 1.00 |  | 0.021 | 1.00 | 0.034 | * |
|  |  | C A | 17 / 28 | 0.56 (0.29; 1.08) | 0.082 |  | 0.50 (0.26; 0.95) |  | * |
|  |  | A A | 0 / 3 | 0.00 (0.00; .) | 0.993 |  |  |  |  |
| FOS | rs7101 | A A | 49 / 50 | 1.00 |  | 1.000 | 1.00 | 0.876 |  |
|  |  | A G | 38 / 36 | 1.08 (0.58; 2.01) | 0.818 |  | 1.05 (0.57; 1.94) |  |  |
|  |  | G G | 4 / 5 | 0.83 (0.21; 3.25) | 0.793 |  |  |  |  |
| GNB1L | rs2904562 | T T | 77 / 81 | 1.00 |  | 0.322 | 1.00 | 0.396 |  |
|  |  | T A | 13 / 10 | 1.33 (0.56; 3.16) | 0.514 |  | 1.44 (0.62; 3.38) |  |  |
|  |  | A A | 1 / 0 | 4004360.21 (0.00; .) | 0.994 |  |  |  |  |
| GNB1L | rs6518585 | G G | 63 / 60 | 1.00 |  | 0.528 | 1.00 | 0.622 |  |
|  |  | G A | 28 / 30 | 0.90 (0.47; 1.72) | 0.739 |  | 0.85 (0.45; 1.62) |  |  |
|  |  | A A | 0 / 1 | 0.00 (0.00; .) | 0.994 |  |  |  |  |
| GPX2 | rs10133054 | G G | 49 / 55 | 1.00 |  | 0.245 | 1.00 | 0.332 |  |
|  |  | G C | 36 / 33 | 1.27 (0.64; 2.50) | 0.492 |  | 1.38 (0.72; 2.62) |  |  |
|  |  | C C | 6 / 3 | 2.17 (0.53; 8.87) | 0.281 |  |  |  |  |
| GPX2 | rs1800669 | A A | 87 / 86 | 1.00 |  | 0.739 | 1.00 | 0.739 |  |
|  |  | A T | 4 / 5 | 0.80 (0.22; 2.98) | 0.739 |  | 0.80 (0.22; 2.98) |  |  |
| GPX2 | rs2296327 | G G | 50 / 52 | 1.00 |  | 0.889 | 1.00 | 0.706 |  |
|  |  | G A | 36 / 33 | 1.23 (0.56; 2.72) | 0.609 |  | 1.15 (0.55; 2.42) |  |  |
|  |  | A A | 5 / 6 | 0.92 (0.27; 3.16) | 0.888 |  |  |  |  |
| GPX3 | rs2042235 | G G | 45 / 52 | 1.00 |  | 0.312 | 1.00 | 0.299 |  |
|  |  | G A | 41 / 35 | 1.36 (0.74; 2.48) | 0.320 |  | 1.37 (0.76; 2.47) |  |  |
|  |  | A A | 5 / 4 | 1.48 (0.38; 5.79) | 0.570 |  |  |  |  |
| GPX3 | rs3805435 | A A | 75 / 75 | 1.00 |  | 1.000 | 1.00 | 1.000 |  |
|  |  | A G | 16 / 16 | 1.00 (0.45; 2.23) | 1.000 |  | 1.00 (0.45; 2.23) |  |  |
| GPX3 | rs3828599 | G G | 53 / 53 | 1.00 |  | 0.706 | 1.00 | 1.000 |  |
|  |  | G A | 32 / 35 | 0.92 (0.50; 1.70) | 0.795 |  | 1.00 (0.55; 1.81) |  |  |
|  |  | A A | 6 / 3 | 1.93 (0.47; 7.92) | 0.362 |  |  |  |  |
| GPX3 | rs8177404 | A A | 2 / 0 | 1.00 |  | 0.993 | 1.00 | 0.991 |  |
|  |  | A G | 89 / 90 | 0.00 (0.00; .) | 0.995 |  | 0.00 (0.00; .) |  |  |
|  |  | G G | 0 / 1 | 0.00 (0.00; .) | 0.994 |  |  |  |  |
| GPX3 | rs8177429 | C C | 81 / 77 | 1.00 |  | 0.436 | 1.00 | 0.374 |  |
|  |  | C G | 9 / 13 | 0.64 (0.25; 1.64) | 0.350 |  | 0.67 (0.27; 1.63) |  |  |
|  |  | G G | 1 / 1 | 1.00 (0.06; 15.99) | 1.000 |  |  |  |  |
| GPX3 | rs8177433 | G G | 63 / 64 | 1.00 |  | 0.769 | 1.00 | 0.866 |  |
|  |  | G A | 25 / 25 | 1.02 (0.52; 2.02) | 0.945 |  | 1.06 (0.55; 2.05) |  |  |
|  |  | A A | 3 / 2 | 1.52 (0.25; 9.25) | 0.653 |  |  |  |  |
| GPX4 | rs3746165 | G G | 24 / 25 | 1.00 |  | 0.920 | 1.00 | 0.876 |  |
|  |  | G A | 48 / 45 | 1.11 (0.57; 2.14) | 0.762 |  | 1.05 (0.57; 1.94) |  |  |
|  |  | A A | 19 / 21 | 0.94 (0.42; 2.08) | 0.873 |  |  |  |  |
| GPX5 | rs13215054 | C C | 65 / 63 | 1.00 |  | 0.572 | 1.00 | 0.752 |  |
|  |  | C A | 25 / 25 | 0.97 (0.51; 1.85) | 0.935 |  | 0.91 (0.49; 1.68) |  |  |
|  |  | A A | 1 / 3 | 0.33 (0.03; 3.20) | 0.340 |  |  |  |  |
| GPX5 | rs2394102 | A A | 78 / 82 | 1.00 |  | 0.436 | 1.00 | 0.374 |  |
|  |  | A G | 12 / 8 | 1.57 (0.61; 4.05) | 0.350 |  | 1.50 (0.61; 3.67) |  |  |
|  |  | G G | 1 / 1 | 1.00 (0.06; 15.99) | 1.000 |  |  |  |  |
| GPX5 | rs380879 | A A | 69 / 73 | 1.00 |  | 0.591 | 1.00 | 0.467 |  |
|  |  | A C | 22 / 17 | 1.31 (0.64; 2.69) | 0.467 |  | 1.31 (0.64; 2.69) |  |  |
|  |  | C C | 0 / 1 | 0.00 (0.00; .) | 0.994 |  |  |  |  |
| GPX5 | rs451774 | A A | 48 / 54 | 1.00 |  | 0.314 | 1.00 | 0.378 |  |
|  |  | A G | 34 / 31 | 1.21 (0.64; 2.30) | 0.551 |  | 1.30 (0.73; 2.33) |  |  |
|  |  | G G | 9 / 6 | 1.67 (0.54; 5.18) | 0.371 |  |  |  |  |
| GPX5 | rs454182 | G G | 50 / 55 | 1.00 |  | 0.367 | 1.00 | 0.467 |  |
|  |  | G C | 32 / 30 | 1.16 (0.62; 2.14) | 0.647 |  | 1.24 (0.70; 2.20) |  |  |
|  |  | C C | 9 / 6 | 1.67 (0.54; 5.20) | 0.374 |  |  |  |  |
| GPX6 | rs1003359 | A A | 45 / 50 | 1.00 |  | 0.509 | 1.00 | 0.476 |  |
|  |  | A G | 37 / 33 | 1.23 (0.67; 2.22) | 0.506 |  | 1.23 (0.70; 2.16) |  |  |
|  |  | G G | 9 / 8 | 1.24 (0.44; 3.52) | 0.687 |  |  |  |  |
| GPX6 | rs1015811 | A A | 50 / 58 | 1.00 |  | 0.461 | 1.00 | 0.250 |  |
|  |  | A G | 38 / 28 | 1.52 (0.84; 2.77) | 0.170 |  | 1.40 (0.79; 2.49) |  |  |
|  |  | G G | 3 / 5 | 0.70 (0.16; 2.99) | 0.628 |  |  |  |  |
| GPX6 | rs1029328 | A A | 76 / 78 | 1.00 |  | 0.715 | 1.00 | 0.683 |  |
|  |  | A G | 14 / 12 | 1.20 (0.52; 2.78) | 0.670 |  | 1.18 (0.53; 2.64) |  |  |
|  |  | G G | 1 / 1 | 1.00 (0.06; 15.99) | 1.000 |  |  |  |  |
| GPX6 | rs11757000 | A A | 69 / 72 | 1.00 |  | 0.648 | 1.00 | 0.591 |  |
|  |  | A G | 20 / 17 | 1.25 (0.59; 2.67) | 0.565 |  | 1.21 (0.60; 2.46) |  |  |
|  |  | G G | 2 / 2 | 1.00 (0.14; 7.10) | 1.000 |  |  |  |  |
| GPX6 | rs2531818 | G G | 80 / 81 | 1.00 |  | 0.671 | 1.00 | 0.819 |  |
|  |  | G C | 10 / 10 | 1.00 (0.40; 2.52) | 1.000 |  | 1.11 (0.45; 2.73) |  |  |
|  |  | C C | 1 / 0 | 4004360.21 (0.00; .) | 0.994 |  |  |  |  |
| GPX6 | rs2859356 | G G | 60 / 65 | 1.00 |  | 0.485 | 1.00 | 0.436 |  |
|  |  | G A | 29 / 24 | 1.30 (0.69; 2.45) | 0.423 |  | 1.28 (0.69; 2.37) |  |  |
|  |  | A A | 2 / 2 | 1.07 (0.15; 7.65) | 0.949 |  |  |  |  |
| GPX7 | rs11591099 | A A | 64 / 65 | 1.00 |  | 0.587 | 1.00 | 0.876 |  |
|  |  | A G | 23 / 25 | 0.93 (0.49; 1.76) | 0.819 |  | 1.05 (0.57; 1.94) |  |  |
|  |  | G G | 4 / 1 | 3.94 (0.44; 35.39) | 0.221 |  |  |  |  |
| GPX7 | rs3753753 | G G | 40 / 47 | 1.00 |  | 0.257 | 1.00 | 0.355 |  |
|  |  | G C | 41 / 38 | 1.20 (0.69; 2.08) | 0.527 |  | 1.28 (0.76; 2.16) |  |  |
|  |  | C C | 10 / 6 | 1.78 (0.63; 5.02) | 0.273 |  |  |  |  |
| GPX7 | rs6588431 | G G | 26 / 28 | 1.00 |  | 0.919 | 1.00 | 0.768 |  |
|  |  | G A | 51 / 46 | 1.18 (0.64; 2.19) | 0.601 |  | 1.09 (0.61; 1.95) |  |  |
|  |  | A A | 14 / 17 | 0.88 (0.38; 2.01) | 0.756 |  |  |  |  |
| GPX7 | rs6588432 | A A | 52 / 60 | 1.00 |  | 0.138 | 1.00 | 0.260 |  |
|  |  | A G | 34 / 30 | 1.26 (0.71; 2.24) | 0.436 |  | 1.38 (0.79; 2.42) |  |  |
|  |  | G G | 5 / 1 | 5.42 (0.63; 46.93) | 0.125 |  |  |  |  |
| GPX7 | rs6671552 | A A | 83 / 74 | 1.00 |  | 0.059 | 1.00 | 0.068 | * |
|  |  | A G | 8 / 16 | 0.47 (0.19; 1.15) | 0.096 |  | 0.44 (0.18; 1.06) |  | * |
|  |  | G G | 0 / 1 | 0.00 (0.00; .) | 0.994 |  |  |  |  |
| GPX7 | rs7529595 | G G | 37 / 42 | 1.00 |  | 0.281 | 1.00 | 0.501 |  |
|  |  | G A | 40 / 41 | 1.04 (0.58; 1.86) | 0.905 |  | 1.20 (0.71; 2.04) |  |  |
|  |  | A A | 14 / 8 | 1.88 (0.74; 4.79) | 0.187 |  |  |  |  |
| GPX7 | rs835342 | A A | 23 / 23 | 1.00 |  | 0.921 | 1.00 | 1.000 |  |
|  |  | A G | 49 / 48 | 1.02 (0.54; 1.95) | 0.948 |  | 1.00 (0.55; 1.83) |  |  |
|  |  | G G | 19 / 20 | 0.95 (0.43; 2.09) | 0.906 |  |  |  |  |
| GPX7 | rs946154 | C C | 34 / 39 | 1.00 |  | 0.381 | 1.00 | 0.508 |  |
|  |  | C G | 44 / 43 | 1.11 (0.64; 1.95) | 0.707 |  | 1.19 (0.71; 2.01) |  |  |
|  |  | G G | 13 / 9 | 1.51 (0.63; 3.64) | 0.360 |  |  |  |  |
| HNRPA3 | rs13029183 | A A | 73 / 72 | 1.00 |  | 1.000 | 1.00 | 0.862 |  |
|  |  | A G | 17 / 19 | 0.88 (0.44; 1.77) | 0.724 |  | 0.94 (0.48; 1.86) |  |  |
|  |  | G G | 1 / 0 | 4004360.21 (0.00; .) | 0.994 |  |  |  |  |
| HNRPA3 | rs2706113 | G G | 77 / 78 | 1.00 |  | 0.828 | 1.00 | 0.828 |  |
|  |  | G A | 14 / 13 | 1.10 (0.47; 2.59) | 0.828 |  | 1.10 (0.47; 2.59) |  |  |
| HS2ST1 | rs11579474 | G G | 82 / 72 | 1.00 |  | 0.049 | 1.00 | 0.056 | * |
|  |  | G A | 9 / 18 | 0.47 (0.20; 1.09) | 0.079 |  | 0.45 (0.19; 1.02) |  | * |
|  |  | A A | 0 / 1 | 0.00 (0.00; .) | 0.994 |  |  |  |  |
| HS2ST1 | rs12139634 | A A | 62 / 59 | 1.00 |  | 0.662 | 1.00 | 0.640 |  |
|  |  | A G | 28 / 31 | 0.86 (0.46; 1.61) | 0.631 |  | 0.86 (0.47; 1.60) |  |  |
|  |  | G G | 1 / 1 | 1.00 (0.06; 15.99) | 1.000 |  |  |  |  |
| HS2ST1 | rs472509 | A A | 75 / 76 | 1.00 |  | 1.000 | 1.00 | 0.842 |  |
|  |  | A G | 15 / 13 | 1.18 (0.53; 2.64) | 0.683 |  | 1.08 (0.49; 2.37) |  |  |
|  |  | G G | 0 / 1 | 0.00 (0.00; .) | 0.994 |  |  |  |  |
| KIAA1324 | rs1052878 | G G | 68 / 79 | 1.00 |  | 0.039 | 1.00 | 0.047 | * |
|  |  | G A | 20 / 11 | 2.13 (0.96; 4.72) | 0.064 |  | 2.22 (1.01; 4.88) |  | * |
|  |  | A A | 3 / 1 | 4.53 (0.44; 47.11) | 0.206 |  |  |  |  |
| LOC390354 | rs11111963 | A A | 28 / 25 | 1.00 |  | 0.606 | 1.00 | 0.631 |  |
|  |  | A G | 56 / 58 | 0.86 (0.46; 1.63) | 0.652 |  | 0.86 (0.46; 1.61) |  |  |
|  |  | G G | 7 / 8 | 0.78 (0.25; 2.42) | 0.665 |  |  |  |  |
| LOC390354 | rs4964735 | G G | 43 / 45 | 1.00 |  | 1.000 | 1.00 | 0.778 |  |
|  |  | G A | 39 / 35 | 1.20 (0.64; 2.25) | 0.564 |  | 1.08 (0.62; 1.89) |  |  |
|  |  | A A | 9 / 11 | 0.81 (0.30; 2.18) | 0.669 |  |  |  |  |
| LOC390354 | rs7310505 | C C | 50 / 60 | 1.00 |  | 0.112 | 1.00 | 0.127 |  |
|  |  | C A | 34 / 27 | 1.53 (0.79; 2.98) | 0.207 |  | 1.63 (0.87; 3.03) |  |  |
|  |  | A A | 6 / 3 | 2.21 (0.55; 8.96) | 0.267 |  |  |  |  |
| LOC729626 | rs12608475 | G G | 26 / 24 | 1.00 |  | 0.918 | 1.00 | 0.732 |  |
|  |  | G A | 41 / 44 | 0.85 (0.41; 1.76) | 0.661 |  | 0.89 (0.45; 1.74) |  |  |
|  |  | A A | 24 / 23 | 0.96 (0.43; 2.14) | 0.913 |  |  |  |  |
| LOC729626 | rs12971544 | A A | 15 / 10 | 1.00 |  | 0.357 | 1.00 | 0.301 |  |
|  |  | A G | 74 / 79 | 0.64 (0.28; 1.49) | 0.298 |  | 0.64 (0.28; 1.49) |  |  |
|  |  | G G | 2 / 2 | 0.72 (0.09; 5.66) | 0.751 |  |  |  |  |
| LOC729626 | rs4801731 | G G | 78 / 83 | 1.00 |  | 0.398 | 1.00 | 0.257 |  |
|  |  | G A | 13 / 7 | 2.00 (0.75; 5.33) | 0.166 |  | 1.71 (0.68; 4.35) |  |  |
|  |  | A A | 0 / 1 | 0.00 (0.00; .) | 0.994 |  |  |  |  |
| LOC729626 | rs8101799 | T T | 54 / 58 | 1.00 |  | 0.308 | 1.00 | 0.572 |  |
|  |  | T A | 29 / 30 | 1.02 (0.56; 1.84) | 0.956 |  | 1.17 (0.67; 2.05) |  |  |
|  |  | A A | 8 / 3 | 2.68 (0.70; 10.20) | 0.149 |  |  |  |  |
| MAN1C1 | rs6694897 | G G | 27 / 35 | 1.00 |  | 0.710 | 1.00 | 0.149 |  |
|  |  | G A | 50 / 37 | 1.90 (0.89; 4.06) | 0.099 |  | 1.73 (0.82; 3.63) |  | * |
|  |  | A A | 14 / 19 | 1.11 (0.41; 3.03) | 0.842 |  |  |  |  |
| MAN1C1 | rs807253 | A A | 50 / 56 | 1.00 |  | 0.289 | 1.00 | 0.332 |  |
|  |  | A G | 36 / 32 | 1.32 (0.67; 2.57) | 0.422 |  | 1.38 (0.72; 2.62) |  |  |
|  |  | G G | 5 / 3 | 1.85 (0.43; 7.96) | 0.408 |  |  |  |  |
| NFE2L2 | rs13005431 | A A | 34 / 30 | 1.00 |  | 0.917 | 1.00 | 0.556 |  |
|  |  | A G | 42 / 49 | 0.75 (0.40; 1.41) | 0.378 |  | 0.84 (0.47; 1.50) |  |  |
|  |  | G G | 13 / 10 | 1.14 (0.47; 2.73) | 0.774 |  |  |  |  |
| NFE2L2 | rs16865105 | A A | 62 / 65 | 1.00 |  | 0.719 | 1.00 | 0.640 |  |
|  |  | A C | 24 / 21 | 1.20 (0.60; 2.39) | 0.602 |  | 1.16 (0.63; 2.14) |  |  |
|  |  | C C | 5 / 5 | 1.02 (0.29; 3.53) | 0.977 |  |  |  |  |
| NFE2L2 | rs1806649 | G G | 49 / 49 | 1.00 |  | 0.829 | 1.00 | 1.000 |  |
|  |  | G A | 33 / 35 | 0.95 (0.54; 1.69) | 0.870 |  | 1.00 (0.58; 1.72) |  |  |
|  |  | A A | 9 / 7 | 1.26 (0.46; 3.49) | 0.658 |  |  |  |  |
| NFE2L2 | rs2364722 | A A | 43 / 36 | 1.00 |  | 0.651 | 1.00 | 0.299 |  |
|  |  | A G | 37 / 47 | 0.66 (0.35; 1.23) | 0.192 |  | 0.73 (0.40; 1.32) |  |  |
|  |  | G G | 11 / 8 | 1.15 (0.41; 3.20) | 0.787 |  |  |  |  |
| NFE2L2 | rs2364725 | A A | 24 / 21 | 1.00 |  | 0.592 | 1.00 | 0.613 |  |
|  |  | A C | 42 / 53 | 0.70 (0.35; 1.43) | 0.329 |  | 0.84 (0.43; 1.64) |  |  |
|  |  | C C | 25 / 17 | 1.31 (0.56; 3.10) | 0.535 |  |  |  |  |
| NFE2L2 | rs2706110 | G G | 59 / 59 | 1.00 |  | 0.879 | 1.00 | 1.000 |  |
|  |  | G A | 31 / 32 | 0.95 (0.51; 1.78) | 0.873 |  | 1.00 (0.54; 1.86) |  |  |
|  |  | A A | 1 / 0 | 4004360.21 (0.00; .) | 0.994 |  |  |  |  |
| NFKB1 | rs10489113 | A A | 63 / 61 | 1.00 |  | 0.468 | 1.00 | 0.732 |  |
|  |  | A G | 27 / 26 | 1.03 (0.51; 2.09) | 0.942 |  | 0.89 (0.45; 1.74) |  |  |
|  |  | G G | 1 / 4 | 0.25 (0.03; 2.26) | 0.218 |  |  |  |  |
| NFKB1 | rs13117745 | G G | 65 / 62 | 1.00 |  | 0.448 | 1.00 | 0.602 |  |
|  |  | G A | 25 / 26 | 0.91 (0.45; 1.84) | 0.786 |  | 0.83 (0.42; 1.65) |  |  |
|  |  | A A | 1 / 3 | 0.33 (0.03; 3.15) | 0.332 |  |  |  |  |
| NFKB1 | rs1609798 | G G | 35 / 43 | 1.00 |  | 0.209 | 1.00 | 0.250 |  |
|  |  | G A | 44 / 40 | 1.32 (0.72; 2.44) | 0.371 |  | 1.40 (0.79; 2.49) |  |  |
|  |  | A A | 12 / 8 | 1.70 (0.67; 4.37) | 0.267 |  |  |  |  |
| NFKB1 | rs230540 | A A | 33 / 43 | 1.00 |  | 0.195 | 1.00 | 0.160 |  |
|  |  | A G | 44 / 37 | 1.48 (0.81; 2.73) | 0.206 |  | 1.50 (0.85; 2.64) |  |  |
|  |  | G G | 14 / 11 | 1.55 (0.65; 3.70) | 0.327 |  |  |  |  |
| NFKB1 | rs230547 | G G | 67 / 75 | 1.00 |  | 0.123 | 1.00 | 0.123 |  |
|  |  | G A | 24 / 16 | 1.89 (0.84; 4.24) | 0.123 |  | 1.89 (0.84; 4.24) |  |  |
| NFKB1 | rs3774934 | G G | 66 / 75 | 1.00 |  | 0.093 | 1.00 | 0.111 | * |
|  |  | G A | 24 / 16 | 1.73 (0.82; 3.63) | 0.149 |  | 1.82 (0.87; 3.79) |  |  |
|  |  | A A | 1 / 0 | 4004360.21 (0.00; .) | 0.994 |  |  |  |  |
| NFKB1 | rs3821958 | A A | 29 / 30 | 1.00 |  | 0.677 | 1.00 | 0.876 |  |
|  |  | A G | 45 / 47 | 0.98 (0.51; 1.89) | 0.955 |  | 1.05 (0.57; 1.94) |  |  |
|  |  | G G | 17 / 14 | 1.24 (0.53; 2.87) | 0.621 |  |  |  |  |
| NFKB1 | rs4648022 | G G | 75 / 72 | 1.00 |  | 0.706 | 1.00 | 0.550 |  |
|  |  | G A | 15 / 19 | 0.71 (0.32; 1.61) | 0.416 |  | 0.79 (0.36; 1.73) |  |  |
|  |  | A A | 1 / 0 | 4004360.21 (0.00; .) | 0.994 |  |  |  |  |
| NFKB1 | rs4648037 | A A | 85 / 78 | 1.00 |  | 0.100 | 1.00 | 0.100 |  |
|  |  | A G | 6 / 13 | 0.42 (0.15; 1.18) | 0.100 |  | 0.42 (0.15; 1.18) |  |  |
| NFKB1 | rs4648127 | G G | 81 / 80 | 1.00 |  | 0.819 | 1.00 | 0.819 |  |
|  |  | G A | 10 / 11 | 0.90 (0.37; 2.22) | 0.819 |  | 0.90 (0.37; 2.22) |  |  |
| NFKB1 | rs4648135 | A A | 85 / 76 | 1.00 |  | 0.099 | 1.00 | 0.048 | * |
|  |  | A G | 5 / 15 | 0.29 (0.09; 0.87) | 0.027 |  | 0.36 (0.13; 0.99) |  | * |
|  |  | G G | 1 / 0 | 4004360.21 (0.00; .) | 0.994 |  |  |  |  |
| NFKB1 | rs7674640 | A A | 25 / 21 | 1.00 |  | 0.559 | 1.00 | 0.494 |  |
|  |  | A G | 47 / 50 | 0.79 (0.40; 1.58) | 0.510 |  | 0.79 (0.40; 1.55) |  |  |
|  |  | G G | 19 / 20 | 0.78 (0.31; 1.96) | 0.590 |  |  |  |  |
| PIB5PA | rs2017301 | G G | 19 / 27 | 1.00 |  | 0.474 | 1.00 | 0.186 |  |
|  |  | G A | 51 / 42 | 1.74 (0.84; 3.60) | 0.135 |  | 1.57 (0.80; 3.07) |  |  |
|  |  | A A | 21 / 22 | 1.32 (0.58; 2.98) | 0.510 |  |  |  |  |
| PIB5PA | rs2240431 | C C | 56 / 61 | 1.00 |  | 0.424 | 1.00 | 0.436 |  |
|  |  | C A | 31 / 27 | 1.26 (0.67; 2.37) | 0.477 |  | 1.28 (0.69; 2.37) |  |  |
|  |  | A A | 4 / 3 | 1.47 (0.32; 6.77) | 0.619 |  |  |  |  |
| PIB5PA | rs2240432 | G G | 42 / 43 | 1.00 |  | 0.906 | 1.00 | 0.876 |  |
|  |  | G A | 41 / 40 | 1.05 (0.56; 1.99) | 0.871 |  | 1.05 (0.57; 1.94) |  |  |
|  |  | A A | 8 / 8 | 1.03 (0.36; 2.92) | 0.956 |  |  |  |  |
| PIB5PA | rs5753466 | G G | 73 / 72 | 1.00 |  | 0.758 | 1.00 | 0.853 |  |
|  |  | G C | 16 / 16 | 0.99 (0.46; 2.13) | 0.969 |  | 0.93 (0.45; 1.93) |  |  |
|  |  | C C | 2 / 3 | 0.67 (0.11; 4.00) | 0.656 |  |  |  |  |
| PIB5PA | rs8137317 | T T | 27 / 24 | 1.00 |  | 0.267 | 1.00 | 0.640 |  |
|  |  | T A | 50 / 45 | 1.00 (0.52; 1.92) | 1.000 |  | 0.86 (0.47; 1.60) |  |  |
|  |  | A A | 14 / 22 | 0.60 (0.26; 1.37) | 0.224 |  |  |  |  |
| PSTK | rs11190 | A A | 28 / 33 | 1.00 |  | 0.118 | 1.00 | 0.413 |  |
|  |  | A C | 45 / 49 | 1.10 (0.55; 2.18) | 0.796 |  | 1.31 (0.69; 2.52) |  |  |
|  |  | C C | 18 / 9 | 2.42 (0.90; 6.47) | 0.079 |  |  |  | * |
| PTGS2 | rs10911902 | G G | 60 / 62 | 1.00 |  | 1.000 | 1.00 | 0.739 |  |
|  |  | G A | 28 / 24 | 1.25 (0.62; 2.53) | 0.533 |  | 1.12 (0.58; 2.15) |  |  |
|  |  | A A | 3 / 5 | 0.63 (0.15; 2.68) | 0.536 |  |  |  |  |
| PTGS2 | rs12042763 | C C | 51 / 51 | 1.00 |  | 0.896 | 1.00 | 1.000 |  |
|  |  | C A | 34 / 35 | 0.98 (0.53; 1.82) | 0.944 |  | 1.00 (0.55; 1.83) |  |  |
|  |  | A A | 6 / 5 | 1.24 (0.32; 4.81) | 0.761 |  |  |  |  |
| PTGS2 | rs12124257 | G G | 77 / 80 | 1.00 |  | 0.655 | 1.00 | 0.493 |  |
|  |  | G C | 13 / 9 | 1.38 (0.55; 3.42) | 0.493 |  | 1.38 (0.55; 3.42) |  |  |
|  |  | C C | 0 / 1 | 0.00 (0.00; .) | 0.994 |  |  |  |  |
| PTGS2 | rs20417 | C C | 51 / 66 | 1.00 |  | 0.027 | 1.00 | 0.025 | * |
|  |  | C G | 38 / 24 | 2.05 (1.07; 3.91) | 0.030 |  | 2.07 (1.09; 3.92) |  | * |
|  |  | G G | 2 / 1 | 2.59 (0.22; 29.81) | 0.447 |  |  |  |  |
| PTGS2 | rs2206593 | G G | 85 / 81 | 1.00 |  | 0.260 | 1.00 | 0.323 |  |
|  |  | G A | 6 / 9 | 0.67 (0.24; 1.87) | 0.442 |  | 0.60 (0.22; 1.65) |  |  |
|  |  | A A | 0 / 1 | 0.00 (0.00; .) | 0.994 |  |  |  |  |
| PTGS2 | rs2745557 | G G | 65 / 65 | 1.00 |  | 0.876 | 1.00 | 1.000 |  |
|  |  | G A | 25 / 24 | 1.04 (0.52; 2.07) | 0.907 |  | 1.00 (0.51; 1.96) |  |  |
|  |  | A A | 1 / 2 | 0.51 (0.05; 5.65) | 0.581 |  |  |  |  |
| PTGS2 | rs4648261 | G G | 85 / 84 | 1.00 |  | 0.782 | 1.00 | 0.782 |  |
|  |  | G A | 6 / 7 | 0.86 (0.29; 2.55) | 0.782 |  | 0.86 (0.29; 2.55) |  |  |
| PTGS2 | rs5275 | A A | 31 / 41 | 1.00 |  | 0.104 | 1.00 | 0.135 |  |
|  |  | A G | 46 / 41 | 1.47 (0.78; 2.81) | 0.237 |  | 1.59 (0.87; 2.91) |  |  |
|  |  | G G | 14 / 9 | 2.07 (0.77; 5.57) | 0.150 |  |  |  |  |
| PTGS2 | rs5277 | C C | 71 / 65 | 1.00 |  | 0.481 | 1.00 | 0.261 |  |
|  |  | C G | 17 / 25 | 0.59 (0.27; 1.29) | 0.183 |  | 0.65 (0.30; 1.38) |  |  |
|  |  | G G | 3 / 1 | 3129688.75 (0.00; .) | 0.992 |  |  |  |  |
| PTGS2 | rs689466 | A A | 64 / 54 | 1.00 |  | 0.069 | 1.00 | 0.109 | * |
|  |  | A G | 26 / 33 | 0.63 (0.32; 1.23) | 0.174 |  | 0.58 (0.30; 1.13) |  |  |
|  |  | G G | 1 / 4 | 0.20 (0.02; 1.89) | 0.161 |  |  |  |  |
| RAB15 | rs11623886 | A A | 38 / 38 | 1.00 |  | 0.900 | 1.00 | 1.000 |  |
|  |  | A G | 41 / 40 | 1.03 (0.49; 2.17) | 0.944 |  | 1.00 (0.49; 2.05) |  |  |
|  |  | G G | 12 / 13 | 0.92 (0.33; 2.53) | 0.865 |  |  |  |  |
| RAB15 | rs2277502 | G G | 62 / 70 | 1.00 |  | 0.084 | 1.00 | 0.162 | * |
|  |  | G A | 24 / 20 | 1.46 (0.69; 3.06) | 0.323 |  | 1.67 (0.82; 3.41) |  |  |
|  |  | A A | 5 / 1 | 5.73 (0.65; 50.20) | 0.115 |  |  |  |  |
| RAB15 | rs2412107 | C C | 63 / 54 | 1.00 |  | 0.139 | 1.00 | 0.163 |  |
|  |  | C A | 24 / 30 | 0.67 (0.35; 1.29) | 0.232 |  | 0.64 (0.34; 1.20) |  |  |
|  |  | A A | 4 / 7 | 0.48 (0.13; 1.70) | 0.251 |  |  |  |  |
| RAB15 | rs3759681 | G G | 43 / 51 | 1.00 |  | 0.101 | 1.00 | 0.198 |  |
|  |  | G A | 38 / 35 | 1.41 (0.73; 2.75) | 0.311 |  | 1.53 (0.80; 2.94) |  |  |
|  |  | A A | 10 / 5 | 2.80 (0.80; 9.83) | 0.109 |  |  |  |  |
| RAB15 | rs7143541 | C C | 78 / 73 | 1.00 |  | 0.339 | 1.00 | 0.339 |  |
|  |  | C A | 13 / 18 | 0.69 (0.32; 1.48) | 0.339 |  | 0.69 (0.32; 1.48) |  |  |
| RAB15 | rs7157667 | A A | 84 / 77 | 1.00 |  | 0.067 | 1.00 | 0.083 | * |
|  |  | A T | 7 / 12 | 0.44 (0.14; 1.44) | 0.177 |  | 0.36 (0.12; 1.14) |  |  |
|  |  | T T | 0 / 2 | 0.00 (0.00; .) | 0.991 |  |  |  |  |
| RELA | rs10896027 | G G | 42 / 36 | 1.00 |  | 0.914 | 1.00 | 0.397 |  |
|  |  | G C | 37 / 48 | 0.68 (0.37; 1.23) | 0.199 |  | 0.79 (0.45; 1.37) |  |  |
|  |  | C C | 12 / 7 | 1.53 (0.55; 4.31) | 0.417 |  |  |  |  |
| RELA | rs11820062 | G G | 26 / 19 | 1.00 |  | 0.695 | 1.00 | 0.253 |  |
|  |  | G A | 41 / 51 | 0.60 (0.29; 1.22) | 0.157 |  | 0.68 (0.35; 1.31) |  |  |
|  |  | A A | 23 / 20 | 0.84 (0.39; 1.83) | 0.662 |  |  |  |  |
| RHOA | rs11716445 | G G | 77 / 79 | 1.00 |  | 0.848 | 1.00 | 0.683 |  |
|  |  | G A | 14 / 11 | 1.30 (0.57; 2.97) | 0.533 |  | 1.18 (0.53; 2.64) |  |  |
|  |  | A A | 0 / 1 | 0.00 (0.00; .) | 0.994 |  |  |  |  |
| RHOA | rs3811699 | A A | 37 / 35 | 1.00 |  | 0.600 | 1.00 | 0.752 |  |
|  |  | A G | 49 / 49 | 0.93 (0.50; 1.73) | 0.811 |  | 0.91 (0.49; 1.68) |  |  |
|  |  | G G | 5 / 7 | 0.67 (0.20; 2.33) | 0.534 |  |  |  |  |
| RHOA | rs7621003 | A A | 26 / 30 | 1.00 |  | 0.523 | 1.00 | 0.538 |  |
|  |  | A G | 47 / 45 | 1.19 (0.63; 2.24) | 0.601 |  | 1.21 (0.66; 2.22) |  |  |
|  |  | G G | 18 / 16 | 1.30 (0.55; 3.08) | 0.558 |  |  |  |  |
| RHOA | rs974495 | G G | 75 / 75 | 1.00 |  |  | 1.00 |  |  |
| RP3-402G11.5 | rs2294400 | G G | 3 / 6 | 1.00 |  | 0.179 | 1.00 | 0.273 |  |
|  |  | G C | 27 / 25 | 2.50 (0.49; 12.89) | 0.273 |  | 2.50 (0.49; 12.89) |  |  |
|  |  | C C | 1 / 0 | 27212455.75 (0.00; .) | 0.996 |  |  |  |  |
| RP3-402G11.5 | rs2294402 | A A | 29 / 26 | 1.00 |  | 0.222 | 1.00 | 0.613 |  |
|  |  | A C | 51 / 47 | 0.92 (0.47; 1.82) | 0.818 |  | 0.84 (0.43; 1.64) |  |  |
|  |  | C C | 11 / 18 | 0.47 (0.17; 1.34) | 0.158 |  |  |  |  |
| RP3-402G11.5 | rs4611754 | G G | 75 / 78 | 1.00 |  | 0.565 | 1.00 | 0.533 |  |
|  |  | G A | 11 / 9 | 1.31 (0.49; 3.52) | 0.597 |  | 1.30 (0.57; 2.97) |  |  |
|  |  | A A | 5 / 4 | 1.29 (0.34; 4.83) | 0.707 |  |  |  |  |
| RP3-402G11.5 | rs5771237 | G G | 26 / 35 | 1.00 |  | 0.361 | 1.00 | 0.143 |  |
|  |  | G C | 53 / 43 | 1.72 (0.87; 3.41) | 0.121 |  | 1.64 (0.85; 3.19) |  |  |
|  |  | C C | 12 / 13 | 1.36 (0.54; 3.46) | 0.515 |  |  |  |  |
| RPL30 | rs1466428 | A A | 47 / 58 | 1.00 |  | 0.123 | 1.00 | 0.082 | * |
|  |  | A T | 35 / 26 | 1.79 (0.90; 3.52) | 0.095 |  | 1.79 (0.93; 3.44) |  | * |
|  |  | T T | 9 / 7 | 1.79 (0.61; 5.24) | 0.287 |  |  |  |  |
| RPL30 | rs2877453 | C C | 35 / 34 | 1.00 |  | 0.584 | 1.00 | 0.873 |  |
|  |  | C A | 42 / 39 | 1.02 (0.53; 1.97) | 0.949 |  | 0.95 (0.51; 1.78) |  |  |
|  |  | A A | 14 / 18 | 0.74 (0.31; 1.81) | 0.514 |  |  |  |  |
| SARS | rs11102964 | A A | 62 / 64 | 1.00 |  | 0.492 | 1.00 | 0.773 |  |
|  |  | A G | 22 / 24 | 0.92 (0.50; 1.72) | 0.800 |  | 1.09 (0.62; 1.92) |  |  |
|  |  | G G | 7 / 3 | 2.31 (0.60; 8.96) | 0.225 |  |  |  |  |
| SARS | rs3120625 | A A | 39 / 45 | 1.00 |  | 0.234 | 1.00 | 0.367 |  |
|  |  | A G | 35 / 35 | 1.13 (0.57; 2.24) | 0.728 |  | 1.32 (0.73; 2.39) |  |  |
|  |  | G G | 17 / 11 | 1.74 (0.74; 4.10) | 0.208 |  |  |  |  |
| SARS | rs4246519 | G G | 30 / 23 | 1.00 |  | 0.491 | 1.00 | 0.265 |  |
|  |  | G A | 35 / 42 | 0.67 (0.34; 1.31) | 0.242 |  | 0.70 (0.37; 1.32) |  |  |
|  |  | A A | 25 / 25 | 0.76 (0.35; 1.65) | 0.481 |  |  |  |  |
| SARS | rs4268379 | G G | 29 / 24 | 1.00 |  | 0.776 | 1.00 | 0.436 |  |
|  |  | G A | 37 / 44 | 0.71 (0.36; 1.40) | 0.320 |  | 0.78 (0.42; 1.45) |  |  |
|  |  | A A | 25 / 23 | 0.91 (0.43; 1.93) | 0.808 |  |  |  |  |
| SARS | rs586254 | A A | 57 / 61 | 1.00 |  | 0.538 | 1.00 | 0.538 |  |
|  |  | G G | 34 / 30 | 1.21 (0.66; 2.22) | 0.538 |  |  |  |  |
| SARS | rs600711 | A A | 74 / 76 | 1.00 |  | 0.335 | 1.00 | 0.696 |  |
|  |  | A G | 13 / 15 | 0.83 (0.36; 1.93) | 0.670 |  | 1.17 (0.54; 2.52) |  |  |
|  |  | G G | 4 / 0 | 10884982.30 (0.00; .) | 0.992 |  |  |  |  |
| SARS | rs611060 | G G | 33 / 32 | 1.00 |  | 0.916 | 1.00 | 0.876 |  |
|  |  | G A | 41 / 42 | 0.95 (0.49; 1.81) | 0.868 |  | 0.95 (0.52; 1.76) |  |  |
|  |  | A A | 17 / 17 | 0.97 (0.41; 2.28) | 0.942 |  |  |  |  |
| SARS | rs611945 | A A | 48 / 48 | 1.00 |  | 0.835 | 1.00 | 1.000 |  |
|  |  | A G | 32 / 34 | 0.92 (0.47; 1.80) | 0.811 |  | 1.00 (0.55; 1.81) |  |  |
|  |  | G G | 11 / 9 | 1.19 (0.48; 2.94) | 0.702 |  |  |  |  |
| SARS | rs685653 | A A | 45 / 57 | 1.00 |  | 0.037 | 1.00 | 0.056 | * |
|  |  | A G | 34 / 27 | 1.77 (0.89; 3.54) | 0.105 |  | 1.92 (0.98; 3.76) |  |  |
|  |  | G G | 12 / 7 | 3.11 (0.89; 10.94) | 0.077 |  |  |  | * |
| SBNO2 | rs2074920 | A A | 24 / 28 | 1.00 |  | 0.474 | 1.00 | 0.506 |  |
|  |  | A G | 43 / 42 | 1.20 (0.59; 2.44) | 0.612 |  | 1.25 (0.65; 2.41) |  |  |
|  |  | G G | 24 / 21 | 1.34 (0.60; 3.02) | 0.478 |  |  |  |  |
| SBNO2 | rs2302109 | A A | 27 / 30 | 1.00 |  | 0.608 | 1.00 | 0.640 |  |
|  |  | A G | 43 / 42 | 1.13 (0.59; 2.17) | 0.715 |  | 1.16 (0.63; 2.14) |  |  |
|  |  | G G | 21 / 19 | 1.23 (0.54; 2.79) | 0.620 |  |  |  |  |
| SBNO2 | rs4807569 | A A | 62 / 48 | 1.00 |  | 0.068 | 1.00 | 0.042 | * |
|  |  | A C | 22 / 34 | 0.53 (0.28; 1.00) | 0.051 |  | 0.53 (0.29; 0.98) |  | * |
|  |  | C C | 6 / 8 | 0.57 (0.18; 1.87) | 0.356 |  |  |  |  |
| SCLY | rs1054640 | G G | 43 / 41 | 1.00 |  | 0.559 |  |  |  |
|  |  | G A | 39 / 38 | 0.96 (0.49; 1.87) | 0.900 |  |  |  |  |
|  |  | A A | 9 / 12 | 0.71 (0.26; 1.91) | 0.497 |  |  |  |  |
| SCLY | rs1128552 | A A | 59 / 54 | 1.00 |  | 0.425 | . (.; .) |  |  |
|  |  | A G | 28 / 32 | 0.78 (0.40; 1.52) | 0.472 |  |  |  |  |
|  |  | G G | 4 / 5 | 0.70 (0.15; 3.17) | 0.643 |  |  |  |  |
| SCLY | rs12993309 | G G | 64 / 57 | 1.00 |  | 0.320 | 1.00 | 0.265 |  |
|  |  | G A | 25 / 32 | 0.68 (0.35; 1.31) | 0.251 |  | 0.70 (0.37; 1.32) |  |  |
|  |  | A A | 2 / 2 | 0.91 (0.13; 6.54) | 0.924 |  |  |  |  |
| SCLY | rs1562337 | G G | 39 / 30 | 1.00 |  | 0.183 | 1.00 | 0.163 |  |
|  |  | G A | 38 / 44 | 0.66 (0.34; 1.25) | 0.201 |  | 0.64 (0.34; 1.20) |  |  |
|  |  | A A | 14 / 17 | 0.58 (0.23; 1.48) | 0.256 |  |  |  |  |
| SCLY | rs2249067 | A A | 66 / 62 | 1.00 |  | 0.662 | 1.00 | 0.528 |  |
|  |  | A G | 23 / 28 | 0.78 (0.42; 1.48) | 0.454 |  | 0.82 (0.44; 1.53) |  |  |
|  |  | G G | 2 / 1 | 1.85 (0.17; 20.62) | 0.618 |  |  |  |  |
| SCLY | rs2249435 | G G | 69 / 66 | 1.00 |  | 0.447 | 1.00 | 0.613 |  |
|  |  | G A | 18 / 19 | 0.89 (0.45; 1.75) | 0.731 |  | 0.84 (0.43; 1.64) |  |  |
|  |  | A A | 1 / 3 | 0.31 (0.03; 3.10) | 0.321 |  |  |  |  |
| SCLY | rs2252416 | G G | 45 / 44 | 1.00 |  | 0.638 | 1.00 | 0.873 |  |
|  |  | G A | 38 / 36 | 1.02 (0.53; 1.98) | 0.945 |  | 0.95 (0.51; 1.78) |  |  |
|  |  | A A | 8 / 11 | 0.71 (0.25; 1.98) | 0.511 |  |  |  |  |
| SCLY | rs3210400 | G G | 23 / 32 | 1.00 |  | 0.117 | 1.00 | 0.111 |  |
|  |  | G A | 47 / 42 | 1.75 (0.83; 3.71) | 0.145 |  | 1.82 (0.87; 3.79) |  |  |
|  |  | A A | 21 / 17 | 2.14 (0.79; 5.84) | 0.136 |  |  |  |  |
| SCLY | rs3739055 | G G | 22 / 30 | 1.00 |  | 0.229 | 1.00 | 0.149 |  |
|  |  | G A | 46 / 40 | 1.72 (0.80; 3.67) | 0.163 |  | 1.73 (0.82; 3.63) |  |  |
|  |  | A A | 23 / 21 | 1.77 (0.67; 4.71) | 0.251 |  |  |  |  |
| SCLY | rs3739057 | C C | 88 / 88 | 1.00 |  | 1.000 | 1.00 | 1.000 |  |
|  |  | C A | 3 / 3 | 1.00 (0.14; 7.10) | 1.000 |  | 1.00 (0.14; 7.10) |  |  |
| SCLY | rs3816348 | G G | 83 / 77 | 1.00 |  | 0.187 | 1.00 | 0.187 |  |
|  |  | A A | 8 / 14 | 0.54 (0.22; 1.35) | 0.187 |  |  |  |  |
| SCLY | rs821483 | A A | 40 / 38 | 1.00 |  | 0.742 | 1.00 | 0.763 |  |
|  |  | A C | 39 / 40 | 0.92 (0.50; 1.70) | 0.797 |  | 0.91 (0.51; 1.65) |  |  |
|  |  | C C | 12 / 13 | 0.87 (0.35; 2.16) | 0.764 |  |  |  |  |
| SECISBP2 | rs3763616 | G G | 50 / 43 | 1.00 |  | 0.753 | 1.00 | 0.355 |  |
|  |  | G A | 30 / 41 | 0.70 (0.40; 1.21) | 0.201 |  | 0.78 (0.46; 1.32) |  |  |
|  |  | A A | 11 / 7 | 1.43 (0.50; 4.05) | 0.502 |  |  |  |  |
| SELENBP1 | rs10788804 | G G | 42 / 49 | 1.00 |  | 0.113 | 1.00 | 0.277 |  |
|  |  | G A | 36 / 36 | 1.25 (0.65; 2.38) | 0.506 |  | 1.41 (0.76; 2.63) |  |  |
|  |  | A A | 13 / 6 | 2.47 (0.87; 7.00) | 0.090 |  |  |  | * |
| SELENBP1 | rs1752380 | A A | 25 / 30 | 1.00 |  | 0.915 | 1.00 | 0.413 |  |
|  |  | A G | 52 / 43 | 1.56 (0.76; 3.18) | 0.224 |  | 1.31 (0.69; 2.52) |  |  |
|  |  | G G | 14 / 18 | 0.93 (0.39; 2.21) | 0.865 |  |  |  |  |
| SELENBP1 | rs17564336 | C C | 48 / 45 | 1.00 |  | 0.455 | 1.00 | 0.631 |  |
|  |  | C A | 36 / 36 | 0.90 (0.47; 1.71) | 0.743 |  | 0.86 (0.46; 1.61) |  |  |
|  |  | A A | 7 / 10 | 0.62 (0.20; 1.90) | 0.403 |  |  |  |  |
| SELENBP1 | rs2769265 | C C | 55 / 60 | 1.00 |  | 0.536 | 1.00 | 0.425 |  |
|  |  | C A | 31 / 26 | 1.36 (0.68; 2.73) | 0.383 |  | 1.29 (0.69; 2.44) |  |  |
|  |  | A A | 5 / 5 | 1.06 (0.31; 3.71) | 0.923 |  |  |  |  |
| SELENBP1 | rs2769270 | G G | 68 / 58 | 1.00 |  | 0.161 | 1.00 | 0.118 |  |
|  |  | G A | 19 / 29 | 0.60 (0.31; 1.13) | 0.115 |  | 0.60 (0.32; 1.14) |  |  |
|  |  | A A | 3 / 3 | 0.71 (0.13; 3.75) | 0.684 |  |  |  |  |
| SELENBP1 | rs2800953 | A A | 58 / 66 | 1.00 |  | 0.080 | 1.00 | 0.230 | * |
|  |  | A G | 26 / 24 | 1.24 (0.66; 2.31) | 0.501 |  | 1.44 (0.79; 2.63) |  |  |
|  |  | G G | 7 / 1 | 7.62 (0.92; 62.97) | 0.060 |  |  |  | * |
| SELENBP1 | rs2864118 | G G | 40 / 38 | 1.00 |  | 0.427 | 1.00 | 0.732 |  |
|  |  | G C | 41 / 38 | 1.01 (0.49; 2.09) | 0.974 |  | 0.89 (0.45; 1.74) |  |  |
|  |  | C C | 10 / 15 | 0.65 (0.26; 1.64) | 0.360 |  |  |  |  |
| SELENBP1 | rs4971036 | G G | 69 / 65 | 1.00 |  | 0.538 | 1.00 | 0.506 |  |
|  |  | G A | 20 / 24 | 0.80 (0.41; 1.55) | 0.504 |  | 0.80 (0.42; 1.54) |  |  |
|  |  | A A | 2 / 2 | 0.85 (0.11; 6.39) | 0.870 |  |  |  |  |
| SELI | rs3820937 | C C | 78 / 83 | 1.00 |  | 0.232 | 1.00 | 0.232 |  |
|  |  | C G | 13 / 8 | 1.83 (0.68; 4.96) | 0.232 |  | 1.83 (0.68; 4.96) |  |  |
| SELI | rs6546932 | A A | 26 / 28 | 1.00 |  | 0.474 | 1.00 | 0.746 |  |
|  |  | A G | 44 / 47 | 1.01 (0.51; 1.99) | 0.975 |  | 1.11 (0.59; 2.10) |  |  |
|  |  | G G | 21 / 16 | 1.37 (0.61; 3.09) | 0.452 |  |  |  |  |
| SELI | rs7588538 | A A | 42 / 42 | 1.00 |  | 0.919 | 1.00 | 1.000 |  |
|  |  | A G | 37 / 38 | 0.96 (0.49; 1.88) | 0.911 |  | 1.00 (0.55; 1.81) |  |  |
|  |  | G G | 12 / 11 | 1.08 (0.46; 2.52) | 0.865 |  |  |  |  |
| SELK | rs1992155 | A A | 31 / 26 | 1.00 |  | 0.687 | 1.00 | 0.476 |  |
|  |  | A G | 42 / 48 | 0.78 (0.43; 1.41) | 0.406 |  | 0.82 (0.46; 1.43) |  |  |
|  |  | G G | 18 / 17 | 0.95 (0.41; 2.21) | 0.904 |  |  |  |  |
| SELK | rs9880056 | A A | 45 / 46 | 1.00 |  | 0.819 | 1.00 | 0.884 |  |
|  |  | A G | 38 / 38 | 1.01 (0.54; 1.88) | 0.977 |  | 1.04 (0.59; 1.85) |  |  |
|  |  | G G | 8 / 7 | 1.20 (0.37; 3.96) | 0.762 |  |  |  |  |
| SELM | rs5753463 | G G | 74 / 79 | 1.00 |  | 0.278 | 1.00 | 0.339 |  |
|  |  | G A | 16 / 12 | 1.36 (0.63; 2.97) | 0.435 |  | 1.46 (0.68; 3.13) |  |  |
|  |  | A A | 1 / 0 | 4004360.21 (0.00; .) | 0.994 |  |  |  |  |
| SELS | rs13329318 | A A | 64 / 66 | 1.00 |  | 0.896 | 1.00 | 0.758 |  |
|  |  | A C | 24 / 21 | 1.15 (0.61; 2.17) | 0.662 |  | 1.10 (0.60; 2.02) |  |  |
|  |  | C C | 3 / 4 | 0.78 (0.17; 3.53) | 0.748 |  |  |  |  |
| SELS | rs2101171 | A A | 51 / 46 | 1.00 |  | 0.190 | 1.00 | 0.447 |  |
|  |  | A G | 37 / 36 | 0.92 (0.49; 1.74) | 0.808 |  | 0.79 (0.43; 1.45) |  |  |
|  |  | G G | 3 / 9 | 0.32 (0.09; 1.23) | 0.097 |  |  |  | * |
| SELS | rs34713741 | G G | 48 / 55 | 1.00 |  | 0.308 | 1.00 | 0.319 |  |
|  |  | G A | 36 / 31 | 1.30 (0.71; 2.36) | 0.392 |  | 1.33 (0.76; 2.35) |  |  |
|  |  | A A | 7 / 5 | 1.53 (0.48; 4.92) | 0.476 |  |  |  |  |
| SELT | rs13097283 | G G | 53 / 35 | 1.00 |  | 0.181 | 1.00 | 0.015 | * |
|  |  | G A | 25 / 48 | 0.34 (0.17; 0.67) | 0.002 |  | 0.49 (0.27; 0.87) |  | * |
|  |  | A A | 13 / 8 | 1.39 (0.50; 3.88) | 0.533 |  |  |  |  |
| SELT | rs1568585 | C C | 41 / 52 | 1.00 |  | 0.355 | 1.00 | 0.097 | * |
|  |  | C A | 45 / 31 | 2.11 (1.06; 4.22) | 0.035 |  | 1.69 (0.91; 3.13) |  | * |
|  |  | A A | 5 / 8 | 0.77 (0.25; 2.44) | 0.662 |  |  |  |  |
| SELT | rs17214746 | A A | 68 / 70 | 1.00 |  | 0.416 | 1.00 | 0.746 |  |
|  |  | A G | 18 / 20 | 0.92 (0.47; 1.82) | 0.818 |  | 1.11 (0.59; 2.10) |  |  |
|  |  | G G | 5 / 1 | 4.94 (0.58; 42.35) | 0.146 |  |  |  |  |
| SELT | rs2204113 | G G | 32 / 23 | 1.00 |  | 0.377 | 1.00 | 0.183 |  |
|  |  | G A | 39 / 48 | 0.62 (0.33; 1.19) | 0.150 |  | 0.67 (0.37; 1.21) |  |  |
|  |  | A A | 20 / 20 | 0.78 (0.35; 1.73) | 0.534 |  |  |  |  |
| SELT | rs2868945 | A A | 72 / 67 | 1.00 |  | 0.640 | 1.00 | 0.371 |  |
|  |  | A G | 16 / 23 | 0.63 (0.30; 1.34) | 0.228 |  | 0.72 (0.35; 1.47) |  |  |
|  |  | G G | 3 / 1 | 2.70 (0.28; 26.28) | 0.393 |  |  |  |  |
| SELT | rs6781861 | A A | 58 / 59 | 1.00 |  | 0.681 | 1.00 | 0.882 |  |
|  |  | A G | 30 / 31 | 1.00 (0.55; 1.81) | 1.000 |  | 1.05 (0.58; 1.88) |  |  |
|  |  | G G | 3 / 1 | 3.00 (0.31; 29.40) | 0.346 |  |  |  |  |
| SELT | rs6802114 | G G | 66 / 55 | 1.00 |  | 0.201 | 1.00 | 0.105 |  |
|  |  | G A | 21 / 33 | 0.57 (0.31; 1.07) | 0.080 |  | 0.61 (0.33; 1.11) |  | * |
|  |  | A A | 4 / 3 | 1.06 (0.23; 4.89) | 0.944 |  |  |  |  |
| SELT | rs9879590 | C C | 37 / 40 | 1.00 |  | 0.751 | 1.00 | 0.648 |  |
|  |  | C G | 42 / 39 | 1.18 (0.62; 2.26) | 0.620 |  | 1.15 (0.63; 2.09) |  |  |
|  |  | G G | 12 / 12 | 1.08 (0.44; 2.62) | 0.869 |  |  |  |  |
| SELV | rs12461523 | A A | 41 / 42 | 1.00 |  | 0.742 | 1.00 | 0.882 |  |
|  |  | A C | 38 / 39 | 0.99 (0.53; 1.86) | 0.986 |  | 1.05 (0.58; 1.88) |  |  |
|  |  | C C | 12 / 10 | 1.25 (0.47; 3.29) | 0.656 |  |  |  |  |
| SELV | rs4802035 | C C | 43 / 46 | 1.00 |  | 0.739 | 1.00 | 0.662 |  |
|  |  | C G | 38 / 35 | 1.15 (0.63; 2.12) | 0.644 |  | 1.14 (0.64; 2.02) |  |  |
|  |  | G G | 10 / 10 | 1.07 (0.39; 2.94) | 0.904 |  |  |  |  |
| SELV | rs4803254 | G G | 55 / 62 | 1.00 |  | 0.493 | 1.00 | 0.288 |  |
|  |  | G A | 34 / 25 | 1.53 (0.82; 2.87) | 0.185 |  | 1.39 (0.76; 2.55) |  |  |
|  |  | A A | 1 / 3 | 0.37 (0.04; 3.57) | 0.388 |  |  |  |  |
| SEMA4D | rs4877077 | A A | 73 / 69 | 1.00 |  | 0.548 | 1.00 | 0.451 |  |
|  |  | A G | 15 / 19 | 0.71 (0.32; 1.61) | 0.415 |  | 0.75 (0.36; 1.59) |  |  |
|  |  | G G | 3 / 3 | 0.95 (0.19; 4.73) | 0.945 |  |  |  |  |
| SEPHS1 | rs17529609 | A A | 60 / 57 | 1.00 |  | 0.580 | 1.00 | 0.613 |  |
|  |  | A G | 28 / 30 | 0.86 (0.43; 1.74) | 0.684 |  | 0.84 (0.43; 1.64) |  |  |
|  |  | G G | 3 / 4 | 0.72 (0.16; 3.26) | 0.669 |  |  |  |  |
| SEPHS1 | rs2181839 | G G | 48 / 55 | 1.00 |  | 0.178 | 1.00 | 0.288 |  |
|  |  | G A | 38 / 34 | 1.29 (0.70; 2.40) | 0.417 |  | 1.39 (0.76; 2.55) |  |  |
|  |  | A A | 5 / 2 | 4.45 (0.49; 40.62) | 0.186 |  |  |  |  |
| SEPHS1 | rs2275129 | G G | 26 / 31 | 1.00 |  | 0.107 | 1.00 | 0.447 |  |
|  |  | G C | 41 / 47 | 1.03 (0.54; 1.95) | 0.941 |  | 1.26 (0.69; 2.31) |  |  |
|  |  | C C | 24 / 13 | 2.25 (0.94; 5.40) | 0.068 |  |  |  | * |
| SEPHS1 | rs3802584 | G G | 85 / 85 | 1.00 |  | 1.000 | 1.00 | 1.000 |  |
|  |  | G A | 6 / 6 | 1.00 (0.29; 3.45) | 1.000 |  | 1.00 (0.29; 3.45) |  |  |
| SEPHS1 | rs7901303 | C C | 32 / 26 | 1.00 |  | 0.257 | 1.00 | 0.356 |  |
|  |  | C A | 44 / 45 | 0.80 (0.42; 1.50) | 0.481 |  | 0.75 (0.41; 1.38) |  |  |
|  |  | A A | 15 / 20 | 0.62 (0.27; 1.43) | 0.260 |  |  |  |  |
| SEPHS1 | rs7923713 | A A | 21 / 21 | 1.00 |  | 0.730 | 1.00 | 1.000 |  |
|  |  | A G | 50 / 53 | 0.95 (0.47; 1.94) | 0.895 |  | 1.00 (0.50; 2.00) |  |  |
|  |  | G G | 20 / 17 | 1.19 (0.48; 2.95) | 0.712 |  |  |  |  |
| SEPHS2 | rs1133238 | G G | 85 / 85 | 1.00 |  | 1.000 | 1.00 | 1.000 |  |
|  |  | G A | 6 / 6 | 1.00 (0.29; 3.45) | 1.000 |  | 1.00 (0.29; 3.45) |  |  |
| SEPHS2 | rs1804600 | C C | 91 / 91 | 1.00 |  |  | 1.00 |  |  |
| SEPN1 | rs1044183 | T T | 76 / 74 | 1.00 |  | 0.683 | 1.00 | 0.683 |  |
|  |  | T A | 15 / 17 | 0.85 (0.38; 1.89) | 0.683 |  | 0.85 (0.38; 1.89) |  |  |
| SEPN1 | rs11247710 | C C | 21 / 23 | 1.00 |  | 1.000 | 1.00 | 0.706 |  |
|  |  | C G | 53 / 49 | 1.18 (0.56; 2.51) | 0.660 |  | 1.15 (0.55; 2.42) |  |  |
|  |  | G G | 17 / 19 | 0.99 (0.36; 2.69) | 0.982 |  |  |  |  |
| SEPN1 | rs11247735 | G G | 28 / 23 | 1.00 |  | 0.559 | 1.00 | 0.413 |  |
|  |  | G A | 45 / 50 | 0.75 (0.39; 1.46) | 0.403 |  | 0.76 (0.40; 1.46) |  |  |
|  |  | A A | 18 / 18 | 0.81 (0.31; 2.08) | 0.659 |  |  |  |  |
| SEPN1 | rs2072749 | A A | 50 / 46 | 1.00 |  | 0.334 | 1.00 | 0.556 |  |
|  |  | A G | 36 / 36 | 0.90 (0.50; 1.63) | 0.724 |  | 0.84 (0.47; 1.50) |  |  |
|  |  | G G | 5 / 9 | 0.47 (0.13; 1.64) | 0.236 |  |  |  |  |
| SEPN1 | rs4659382 | G G | 51 / 45 | 1.00 |  | 0.189 | 1.00 | 0.367 |  |
|  |  | G C | 35 / 36 | 0.83 (0.45; 1.55) | 0.564 |  | 0.76 (0.42; 1.38) |  |  |
|  |  | C C | 5 / 10 | 0.40 (0.12; 1.37) | 0.146 |  |  |  |  |
| SEPN1 | rs6872 | G G | 47 / 41 | 1.00 |  | 0.121 | 1.00 | 0.356 |  |
|  |  | G A | 44 / 44 | 0.95 (0.50; 1.81) | 0.869 |  | 0.75 (0.41; 1.38) |  |  |
|  |  | A A | 0 / 6 | 0.00 (0.00; .) | 0.990 |  |  |  |  |
| SEPN1 | rs807245 | G G | 55 / 59 | 1.00 |  | 0.433 | 1.00 | 0.528 |  |
|  |  | G A | 31 / 29 | 1.16 (0.61; 2.22) | 0.650 |  | 1.22 (0.66; 2.28) |  |  |
|  |  | A A | 5 / 3 | 1.76 (0.41; 7.55) | 0.444 |  |  |  |  |
| SEPP1 | rs12519532 | G G | 43 / 44 | 1.00 |  | 0.809 | 1.00 | 0.886 |  |
|  |  | G A | 44 / 40 | 1.09 (0.62; 1.93) | 0.761 |  | 1.04 (0.60; 1.82) |  |  |
|  |  | A A | 4 / 7 | 0.60 (0.17; 2.17) | 0.441 |  |  |  |  |
| SEPP1 | rs13153784 | G G | 45 / 51 | 1.00 |  | 0.468 | 1.00 | 0.397 |  |
|  |  | G C | 42 / 36 | 1.29 (0.73; 2.30) | 0.382 |  | 1.27 (0.73; 2.23) |  |  |
|  |  | C C | 4 / 4 | 1.10 (0.27; 4.49) | 0.893 |  |  |  |  |
| SEPP1 | rs13171019 | A A | 66 / 63 | 1.00 |  | 0.769 | 1.00 | 0.622 |  |
|  |  | A G | 23 / 27 | 0.79 (0.40; 1.55) | 0.494 |  | 0.85 (0.45; 1.62) |  |  |
|  |  | G G | 2 / 1 | 2.00 (0.18; 22.05) | 0.572 |  |  |  |  |
| SEPP1 | rs230813 | C C | 21 / 29 | 1.00 |  | 0.514 | 1.00 | 0.174 |  |
|  |  | C G | 52 / 42 | 1.74 (0.85; 3.59) | 0.131 |  | 1.62 (0.81; 3.23) |  |  |
|  |  | G G | 18 / 20 | 1.32 (0.55; 3.14) | 0.538 |  |  |  |  |
| SEPP1 | rs2973011 | A A | 21 / 29 | 1.00 |  | 0.584 | 1.00 | 0.174 |  |
|  |  | A G | 52 / 41 | 1.78 (0.87; 3.68) | 0.117 |  | 1.62 (0.81; 3.23) |  |  |
|  |  | G G | 18 / 21 | 1.24 (0.52; 2.98) | 0.632 |  |  |  |  |
| SEPP1 | rs3877899 | G G | 54 / 56 | 1.00 |  | 0.803 | 1.00 | 0.768 |  |
|  |  | G A | 34 / 32 | 1.11 (0.59; 2.06) | 0.752 |  | 1.09 (0.61; 1.95) |  |  |
|  |  | A A | 3 / 3 | 1.00 (0.20; 4.96) | 1.000 |  |  |  |  |
| SEPP1 | rs4866800 | C C | 24 / 30 | 1.00 |  | 1.000 | 1.00 | 0.306 |  |
|  |  | C A | 54 / 42 | 1.65 (0.80; 3.36) | 0.173 |  | 1.43 (0.72; 2.83) |  |  |
|  |  | A A | 13 / 19 | 0.92 (0.37; 2.29) | 0.856 |  |  |  |  |
| SEPP1 | rs6413428 | A A | 54 / 56 | 1.00 |  | 0.803 | 1.00 | 0.768 |  |
|  |  | A G | 34 / 32 | 1.11 (0.59; 2.06) | 0.752 |  | 1.09 (0.61; 1.95) |  |  |
|  |  | G G | 3 / 3 | 1.00 (0.20; 4.96) | 1.000 |  |  |  |  |
| SEPP1 | rs7579 | G G | 45 / 39 | 1.00 |  | 0.247 | 1.00 | 0.415 |  |
|  |  | G A | 42 / 44 | 0.84 (0.49; 1.44) | 0.520 |  | 0.80 (0.47; 1.37) |  |  |
|  |  | A A | 4 / 8 | 0.44 (0.13; 1.56) | 0.205 |  |  |  |  |
| SEPSECS | rs10026220 | G G | 83 / 79 | 1.00 |  | 0.533 | 1.00 | 0.374 |  |
|  |  | G A | 7 / 12 | 0.58 (0.23; 1.48) | 0.257 |  | 0.67 (0.27; 1.63) |  |  |
|  |  | A A | 1 / 0 | 4004360.21 (0.00; .) | 0.994 |  |  |  |  |
| SEPSECS | rs13139513 | G G | 24 / 27 | 1.00 |  | 0.518 | 1.00 | 0.591 |  |
|  |  | G A | 45 / 45 | 1.17 (0.56; 2.45) | 0.686 |  | 1.21 (0.60; 2.46) |  |  |
|  |  | A A | 22 / 19 | 1.33 (0.57; 3.11) | 0.518 |  |  |  |  |
| SEPSECS | rs16876882 | G G | 72 / 74 | 1.00 |  | 0.550 | 1.00 | 0.670 |  |
|  |  | G A | 18 / 17 | 1.10 (0.47; 2.59) | 0.827 |  | 1.20 (0.52; 2.78) |  |  |
|  |  | A A | 1 / 0 | 4004360.21 (0.00; .) | 0.994 |  |  |  |  |
| SEPSECS | rs17480524 | A A | 81 / 81 | 1.00 |  | 1.000 | 1.00 | 1.000 |  |
|  |  | A G | 10 / 10 | 1.00 (0.40; 2.52) | 1.000 |  | 1.00 (0.40; 2.52) |  |  |
| SEPSECS | rs3756207 | A A | 64 / 62 | 1.00 |  | 1.000 | 1.00 | 0.768 |  |
|  |  | A G | 25 / 29 | 0.88 (0.49; 1.57) | 0.655 |  | 0.92 (0.51; 1.64) |  |  |
|  |  | G G | 2 / 0 | 3750073.52 (0.00; .) | 0.992 |  |  |  |  |
| SEPSECS | rs3796791 | G G | 34 / 29 | 1.00 |  | 0.266 | 1.00 | 0.493 |  |
|  |  | G A | 46 / 45 | 0.90 (0.51; 1.59) | 0.720 |  | 0.83 (0.48; 1.42) |  |  |
|  |  | A A | 11 / 17 | 0.57 (0.23; 1.38) | 0.209 |  |  |  |  |
| SEPSECS | rs3796795 | C C | 68 / 65 | 1.00 |  | 0.769 | 1.00 | 0.613 |  |
|  |  | C A | 20 / 24 | 0.80 (0.41; 1.60) | 0.533 |  | 0.84 (0.43; 1.64) |  |  |
|  |  | A A | 3 / 2 | 1.38 (0.23; 8.42) | 0.730 |  |  |  |  |
| SEPW1 | rs10412662 | A A | 65 / 64 | 1.00 |  | 0.865 | 1.00 | 0.866 |  |
|  |  | A C | 1 / 1 | 0.97 (0.06; 15.85) | 0.984 |  | 0.94 (0.49; 1.83) |  |  |
|  |  | C C | 21 / 22 | 0.94 (0.48; 1.84) | 0.865 |  |  |  |  |
| SEPW1 | rs11083897 | A A | 36 / 39 | 1.00 |  | 0.492 | 1.00 | 0.662 |  |
|  |  | A G | 45 / 45 | 1.09 (0.61; 1.96) | 0.772 |  | 1.14 (0.64; 2.02) |  |  |
|  |  | G G | 10 / 7 | 1.51 (0.54; 4.27) | 0.436 |  |  |  |  |
| SEPW1 | rs2042286 | G G | 34 / 29 | 1.00 |  | 0.829 | 1.00 | 0.447 |  |
|  |  | G A | 40 / 48 | 0.74 (0.39; 1.38) | 0.336 |  | 0.79 (0.43; 1.45) |  |  |
|  |  | A A | 17 / 14 | 1.05 (0.43; 2.54) | 0.921 |  |  |  |  |
| SMTN | rs2074736 | A A | 42 / 52 | 1.00 |  | 0.353 | 1.00 | 0.176 |  |
|  |  | A G | 41 / 30 | 1.59 (0.88; 2.86) | 0.123 |  | 1.46 (0.85; 2.50) |  |  |
|  |  | G G | 8 / 9 | 1.04 (0.39; 2.78) | 0.931 |  |  |  |  |
| SMTN | rs2074738 | C C | 71 / 73 | 1.00 |  | 0.401 | 1.00 | 0.696 |  |
|  |  | C G | 17 / 18 | 0.92 (0.40; 2.08) | 0.835 |  | 1.17 (0.54; 2.52) |  |  |
|  |  | G G | 3 / 0 | 10884982.30 (0.00; .) | 0.993 |  |  |  |  |
| SMTN | rs8135353 | C C | 29 / 32 | 1.00 |  | 0.667 | 1.00 | 0.648 |  |
|  |  | C A | 52 / 42 | 1.38 (0.72; 2.61) | 0.330 |  | 1.15 (0.63; 2.09) |  |  |
|  |  | A A | 10 / 17 | 0.64 (0.25; 1.61) | 0.340 |  |  |  |  |
| SMTN | rs917208 | G G | 74 / 75 | 1.00 |  | 1.000 | 1.00 | 0.848 |  |
|  |  | G A | 16 / 14 | 1.17 (0.54; 2.52) | 0.695 |  | 1.08 (0.51; 2.29) |  |  |
|  |  | A A | 0 / 1 | 0.00 (0.00; .) | 0.994 |  |  |  |  |
| SNRPA1 | rs10152335 | G G | 36 / 45 | 1.00 |  | 0.255 | 1.00 | 0.173 |  |
|  |  | G A | 46 / 38 | 1.58 (0.82; 3.03) | 0.174 |  | 1.53 (0.83; 2.82) |  |  |
|  |  | A A | 9 / 8 | 1.37 (0.48; 3.94) | 0.557 |  |  |  |  |
| Sep-15 | rs1407131 | A A | 80 / 66 | 1.00 |  | 0.015 | 1.00 | 0.017 | * |
|  |  | A G | 11 / 24 | 0.41 (0.19; 0.89) | 0.024 |  | 0.39 (0.18; 0.85) |  | * |
|  |  | G G | 0 / 1 | 0.00 (0.00; .) | 0.994 |  |  |  |  |
| Sep-15 | rs17357686 | G G | 84 / 80 | 1.00 |  | 0.350 | 1.00 | 0.350 |  |
|  |  | G A | 7 / 11 | 0.64 (0.25; 1.64) | 0.350 |  | 0.64 (0.25; 1.64) |  |  |
| Sep-15 | rs2783974 | G G | 82 / 68 | 1.00 |  | 0.011 | 1.00 | 0.012 | * |
|  |  | G A | 9 / 22 | 0.35 (0.15; 0.83) | 0.017 |  | 0.33 (0.14; 0.78) |  | * |
|  |  | A A | 0 / 1 | 0.00 (0.00; .) | 0.994 |  |  |  |  |
| Sep-15 | rs492745 | A A | 77 / 77 | 1.00 |  | 0.848 | 1.00 | 1.000 |  |
|  |  | A G | 14 / 13 | 1.09 (0.48; 2.47) | 0.835 |  | 1.00 (0.45; 2.23) |  |  |
|  |  | G G | 0 / 1 | 0.00 (0.00; .) | 0.994 |  |  |  |  |
| Sep-15 | rs526985 | G G | 68 / 59 | 1.00 |  | 0.171 | 1.00 | 0.143 |  |
|  |  | G A | 21 / 29 | 0.60 (0.29; 1.23) | 0.162 |  | 0.61 (0.31; 1.18) |  |  |
|  |  | A A | 2 / 3 | 0.67 (0.11; 3.99) | 0.657 |  |  |  |  |
| Sep-15 | rs540049 | G G | 66 / 56 | 1.00 |  | 0.077 | 1.00 | 0.109 | * |
|  |  | G A | 23 / 29 | 0.66 (0.32; 1.34) | 0.251 |  | 0.58 (0.30; 1.13) |  |  |
|  |  | A A | 2 / 6 | 0.32 (0.06; 1.57) | 0.159 |  |  |  |  |
| Sep-15 | rs561104 | A A | 23 / 29 | 1.00 |  | 0.094 | 1.00 | 0.332 | * |
|  |  | A G | 48 / 50 | 1.25 (0.65; 2.41) | 0.507 |  | 1.38 (0.72; 2.62) |  |  |
|  |  | G G | 20 / 12 | 2.76 (0.94; 8.15) | 0.066 |  |  |  | * |
| Sep-15 | rs565469 | G G | 66 / 56 | 1.00 |  | 0.077 | 1.00 | 0.109 | * |
|  |  | G A | 23 / 29 | 0.66 (0.32; 1.34) | 0.251 |  | 0.58 (0.30; 1.13) |  |  |
|  |  | A A | 2 / 6 | 0.32 (0.06; 1.57) | 0.159 |  |  |  |  |
| Sep-15 | rs572464 | C C | 67 / 75 | 1.00 |  | 0.124 | 1.00 | 0.149 |  |
|  |  | C G | 23 / 16 | 1.64 (0.77; 3.47) | 0.198 |  | 1.73 (0.82; 3.63) |  |  |
|  |  | G G | 1 / 0 | 4004360.21 (0.00; .) | 0.994 |  |  |  |  |
| Sep-15 | rs5845 | G G | 66 / 50 | 1.00 |  | 0.015 | 1.00 | 0.014 | * |
|  |  | G A | 23 / 35 | 0.46 (0.22; 0.95) | 0.036 |  | 0.43 (0.22; 0.84) |  | * |
|  |  | A A | 2 / 6 | 0.30 (0.06; 1.50) | 0.142 |  |  |  |  |
| Sep-15 | rs5859 | A A | 84 / 85 | 1.00 |  | 0.657 | 1.00 | 0.657 |  |
|  |  | A G | 4 / 3 | 1.50 (0.25; 8.98) | 0.657 |  | 1.50 (0.25; 8.98) |  |  |
| Sep-15 | rs9433110 | G G | 77 / 77 | 1.00 |  | 0.848 | 1.00 | 1.000 |  |
|  |  | G A | 14 / 13 | 1.09 (0.48; 2.47) | 0.835 |  | 1.00 (0.45; 2.23) |  |  |
|  |  | A A | 0 / 1 | 0.00 (0.00; .) | 0.994 |  |  |  |  |
| TNIP1 | rs10051105 | A A | 80 / 82 | 1.00 |  | 0.515 | 1.00 | 0.638 |  |
|  |  | A G | 10 / 9 | 1.13 (0.43; 2.92) | 0.809 |  | 1.25 (0.49; 3.17) |  |  |
|  |  | G G | 1 / 0 | 4004360.21 (0.00; .) | 0.994 |  |  |  |  |
| TNIP1 | rs11747926 | G G | 63 / 65 | 1.00 |  | 0.407 | 1.00 | 0.752 |  |
|  |  | G A | 24 / 26 | 0.90 (0.47; 1.72) | 0.739 |  | 1.11 (0.59; 2.06) |  |  |
|  |  | A A | 4 / 0 | 10884982.30 (0.00; .) | 0.992 |  |  |  |  |
| TNIP1 | rs2287720 | A A | 32 / 33 | 1.00 |  | 0.408 | 1.00 | 0.879 |  |
|  |  | A G | 46 / 51 | 0.94 (0.51; 1.74) | 0.844 |  | 1.05 (0.58; 1.91) |  |  |
|  |  | G G | 12 / 6 | 2.12 (0.69; 6.51) | 0.191 |  |  |  |  |
| TNIP1 | rs3763009 | G G | 42 / 36 | 1.00 |  | 0.320 | 1.00 | 0.332 |  |
|  |  | G A | 37 / 40 | 0.76 (0.38; 1.49) | 0.419 |  | 0.73 (0.38; 1.39) |  |  |
|  |  | A A | 12 / 15 | 0.66 (0.26; 1.64) | 0.365 |  |  |  |  |
| TNIP1 | rs4958876 | C C | 71 / 71 | 1.00 |  | 0.758 | 1.00 | 1.000 |  |
|  |  | C A | 18 / 20 | 0.89 (0.45; 1.74) | 0.732 |  | 1.00 (0.52; 1.92) |  |  |
|  |  | A A | 2 / 0 | 4004360.21 (0.00; .) | 0.991 |  |  |  |  |
| TNIP1 | rs8177834 | G G | 73 / 77 | 1.00 |  | 0.294 | 1.00 | 0.416 |  |
|  |  | G A | 15 / 13 | 1.30 (0.57; 2.99) | 0.531 |  | 1.40 (0.62; 3.15) |  |  |
|  |  | A A | 3 / 1 | 3.44 (0.34; 34.65) | 0.294 |  |  |  |  |
| TRABD | rs2272845 | G G | 52 / 59 | 1.00 |  | 0.219 | 1.00 | 0.329 |  |
|  |  | G C | 31 / 28 | 1.21 (0.68; 2.17) | 0.521 |  | 1.32 (0.76; 2.29) |  |  |
|  |  | C C | 8 / 4 | 2.13 (0.63; 7.22) | 0.223 |  |  |  |  |
| TRABD | rs3747941 | G G | 62 / 64 | 1.00 |  | 0.594 | 1.00 | 0.752 |  |
|  |  | G A | 25 / 25 | 1.02 (0.53; 1.96) | 0.956 |  | 1.11 (0.59; 2.06) |  |  |
|  |  | A A | 4 / 2 | 2.01 (0.37; 10.99) | 0.423 |  |  |  |  |
| TRABD | rs6712 | C C | 67 / 68 | 1.00 |  | 0.739 | 1.00 | 0.862 |  |
|  |  | C G | 23 / 23 | 1.00 (0.50; 2.00) | 1.000 |  | 1.06 (0.54; 2.10) |  |  |
|  |  | G G | 1 / 0 | 4004360.21 (0.00; .) | 0.994 |  |  |  |  |
| TRABD | rs732710 | G G | 55 / 58 | 1.00 |  | 0.487 | 1.00 | 0.662 |  |
|  |  | G A | 29 / 29 | 1.04 (0.56; 1.92) | 0.898 |  | 1.14 (0.64; 2.02) |  |  |
|  |  | A A | 7 / 4 | 1.77 (0.51; 6.11) | 0.367 |  |  |  |  |
| TRABD | rs735119 | C C | 49 / 57 | 1.00 |  | 0.164 | 1.00 | 0.209 |  |
|  |  | C A | 36 / 31 | 1.40 (0.72; 2.72) | 0.318 |  | 1.50 (0.80; 2.82) |  |  |
|  |  | A A | 6 / 3 | 2.25 (0.55; 9.21) | 0.260 |  |  |  |  |
| TRSPAP1 | rs1342086 | G G | 47 / 37 | 1.00 |  | 0.229 | 1.00 | 0.127 |  |
|  |  | G C | 36 / 46 | 0.60 (0.31; 1.13) | 0.115 |  | 0.62 (0.33; 1.15) |  |  |
|  |  | C C | 8 / 8 | 0.74 (0.24; 2.28) | 0.604 |  |  |  |  |
| TRSPAP1 | rs9426420 | G G | 73 / 68 | 1.00 |  | 0.279 | 1.00 | 0.356 |  |
|  |  | G A | 17 / 20 | 0.79 (0.36; 1.73) | 0.550 |  | 0.71 (0.34; 1.48) |  |  |
|  |  | A A | 1 / 3 | 0.33 (0.04; 3.21) | 0.341 |  |  |  |  |
| TUBGCP6 | rs11703226 | G G | 15 / 20 | 1.00 |  | 0.185 | 1.00 | 0.339 |  |
|  |  | G A | 66 / 65 | 1.36 (0.63; 2.97) | 0.435 |  | 1.46 (0.68; 3.13) |  |  |
|  |  | A A | 10 / 6 | 2.53 (0.66; 9.71) | 0.176 |  |  |  |  |
| TUBGCP6 | rs5771107 | T T | 67 / 68 | 1.00 |  | 0.580 | 1.00 | 0.866 |  |
|  |  | T A | 19 / 21 | 0.93 (0.46; 1.88) | 0.838 |  | 1.06 (0.55; 2.05) |  |  |
|  |  | A A | 5 / 2 | 2.45 (0.47; 12.77) | 0.288 |  |  |  |  |
| TUBGCP6 | rs5771242 | G G | 51 / 56 | 1.00 |  | 0.401 | 1.00 | 0.447 |  |
|  |  | G A | 34 / 31 | 1.21 (0.63; 2.31) | 0.574 |  | 1.26 (0.69; 2.31) |  |  |
|  |  | A A | 6 / 4 | 1.56 (0.44; 5.56) | 0.495 |  |  |  |  |
| TXN | rs10759402 | A A | 54 / 58 | 1.00 |  | 0.299 | 1.00 | 0.572 |  |
|  |  | A G | 6 / 11 | 0.50 (0.17; 1.46) | 0.206 |  | 1.17 (0.67; 2.05) |  |  |
|  |  | G G | 30 / 21 | 1.69 (0.85; 3.36) | 0.133 |  |  |  |  |
| TXN | rs10980282 | T T | 42 / 41 | 1.00 |  | 0.913 | 1.00 | 0.889 |  |
|  |  | T A | 40 / 43 | 0.90 (0.50; 1.62) | 0.734 |  | 0.96 (0.56; 1.67) |  |  |
|  |  | A A | 9 / 7 | 1.29 (0.44; 3.78) | 0.648 |  |  |  |  |
| TXN | rs16914868 | G G | 69 / 75 | 1.00 |  | 0.292 | 1.00 | 0.292 |  |
|  |  | G A | 22 / 16 | 1.46 (0.72; 2.96) | 0.292 |  | 1.46 (0.72; 2.96) |  |  |
| TXN | rs2026312 | T T | 32 / 30 | 1.00 |  | 0.363 | 1.00 | 0.752 |  |
|  |  | T A | 46 / 41 | 1.13 (0.56; 2.28) | 0.736 |  | 0.91 (0.49; 1.68) |  |  |
|  |  | A A | 13 / 20 | 0.62 (0.27; 1.44) | 0.265 |  |  |  |  |
| TXN | rs2026314 | C C | 40 / 40 | 1.00 |  | 0.748 | 1.00 | 1.000 |  |
|  |  | C A | 40 / 37 | 1.09 (0.58; 2.05) | 0.799 |  | 1.00 (0.55; 1.81) |  |  |
|  |  | A A | 11 / 14 | 0.78 (0.31; 1.95) | 0.595 |  |  |  |  |
| TXN | rs2301241 | A A | 28 / 33 | 1.00 |  | 0.266 | 1.00 | 0.467 |  |
|  |  | A G | 48 / 48 | 1.15 (0.64; 2.09) | 0.635 |  | 1.24 (0.70; 2.20) |  |  |
|  |  | G G | 15 / 10 | 1.88 (0.69; 5.14) | 0.220 |  |  |  |  |
| TXN | rs2301242 | A A | 41 / 40 | 1.00 |  | 0.559 | 1.00 | 0.893 |  |
|  |  | A T | 39 / 46 | 0.91 (0.53; 1.56) | 0.733 |  | 0.96 (0.57; 1.64) |  |  |
|  |  | T T | 11 / 5 | 2.80 (0.71; 10.97) | 0.140 |  |  |  |  |
| TXN | rs2418076 | G G | 56 / 46 | 1.00 |  | 0.075 | 1.00 | 0.118 | * |
|  |  | G A | 29 / 33 | 0.67 (0.34; 1.33) | 0.256 |  | 0.60 (0.32; 1.14) |  |  |
|  |  | A A | 6 / 12 | 0.42 (0.15; 1.18) | 0.098 |  |  |  | * |
| TXN | rs4135162 | C C | 89 / 85 | 1.00 |  | 0.179 | 1.00 | 0.179 |  |
|  |  | C G | 2 / 6 | 0.33 (0.07; 1.65) | 0.179 |  | 0.33 (0.07; 1.65) |  |  |
| TXN | rs4135163 | C C | 87 / 87 | 1.00 |  | 1.000 | 1.00 | 1.000 |  |
|  |  | A A | 3 / 3 | 1.00 (0.14; 7.10) | 1.000 |  |  |  |  |
| TXN | rs4135165 | G G | 72 / 68 | 1.00 |  | 0.746 | 1.00 | 0.481 |  |
|  |  | G A | 17 / 23 | 0.67 (0.32; 1.38) | 0.277 |  | 0.78 (0.39; 1.56) |  |  |
|  |  | A A | 2 / 0 | 4004360.21 (0.00; .) | 0.991 |  |  |  |  |
| TXN | rs4135168 | A A | 47 / 55 | 1.00 |  | 0.509 | 1.00 | 0.230 |  |
|  |  | A G | 42 / 31 | 1.56 (0.84; 2.89) | 0.157 |  | 1.44 (0.79; 2.63) |  |  |
|  |  | G G | 2 / 5 | 0.51 (0.10; 2.74) | 0.433 |  |  |  |  |
| TXN | rs4135182 | A A | 74 / 79 | 1.00 |  | 0.452 | 1.00 | 0.321 |  |
|  |  | A C | 17 / 11 | 1.67 (0.73; 3.81) | 0.226 |  | 1.50 (0.67; 3.34) |  |  |
|  |  | C C | 0 / 1 | 0.00 (0.00; .) | 0.994 |  |  |  |  |
| TXN | rs4135192 | A A | 39 / 37 | 1.00 |  | 0.671 | 1.00 | 0.763 |  |
|  |  | A G | 40 / 40 | 0.96 (0.50; 1.83) | 0.895 |  | 0.91 (0.51; 1.65) |  |  |
|  |  | G G | 12 / 14 | 0.80 (0.32; 2.01) | 0.642 |  |  |  |  |
| TXN | rs4135203 | G G | 56 / 55 | 1.00 |  | 0.790 | 1.00 | 0.876 |  |
|  |  | G C | 31 / 31 | 0.98 (0.52; 1.84) | 0.945 |  | 0.95 (0.52; 1.76) |  |  |
|  |  | C C | 4 / 5 | 0.74 (0.16; 3.40) | 0.702 |  |  |  |  |
| TXN | rs4135212 | G G | 64 / 67 | 1.00 |  | 0.547 | 1.00 | 0.622 |  |
|  |  | G A | 25 / 23 | 1.14 (0.59; 2.20) | 0.696 |  | 1.18 (0.62; 2.25) |  |  |
|  |  | A A | 2 / 1 | 2.09 (0.19; 23.32) | 0.549 |  |  |  |  |
| TXN | rs4135215 | A A | 70 / 67 | 1.00 |  | 0.387 | 1.00 | 0.578 |  |
|  |  | A G | 21 / 22 | 0.87 (0.41; 1.82) | 0.706 |  | 0.81 (0.39; 1.69) |  |  |
|  |  | G G | 0 / 2 | 0.00 (0.00; .) | 0.991 |  |  |  |  |
| TXN | rs4135220 | C C | 24 / 23 | 1.00 |  | 0.920 | 1.00 | 0.870 |  |
|  |  | C A | 43 / 46 | 0.91 (0.46; 1.79) | 0.777 |  | 0.95 (0.50; 1.81) |  |  |
|  |  | A A | 24 / 22 | 1.04 (0.47; 2.29) | 0.925 |  |  |  |  |
| TXN | rs4135223 | A A | 80 / 79 | 1.00 |  | 0.819 | 1.00 | 0.819 |  |
|  |  | A C | 11 / 12 | 0.90 (0.37; 2.22) | 0.819 |  | 0.90 (0.37; 2.22) |  |  |
| TXN | rs749384 | G G | 52 / 51 | 1.00 |  | 0.485 | 1.00 | 0.866 |  |
|  |  | G A | 36 / 33 | 1.06 (0.54; 2.12) | 0.861 |  | 0.94 (0.49; 1.83) |  |  |
|  |  | A A | 3 / 7 | 0.34 (0.07; 1.76) | 0.201 |  |  |  |  |
| TXNRD1 | rs10735396 | A A | 73 / 79 | 1.00 |  | 0.223 | 1.00 | 0.277 |  |
|  |  | A G | 15 / 11 | 1.36 (0.63; 2.97) | 0.435 |  | 1.50 (0.72; 3.11) |  |  |
|  |  | G G | 3 / 1 | 3.00 (0.31; 28.84) | 0.341 |  |  |  |  |
| TXNRD1 | rs10778325 | A A | 49 / 61 | 1.00 |  | 0.086 | 1.00 | 0.056 | * |
|  |  | A G | 36 / 25 | 1.97 (0.98; 4.00) | 0.059 |  | 1.92 (0.98; 3.76) |  | * |
|  |  | G G | 5 / 4 | 1.63 (0.35; 7.51) | 0.533 |  |  |  |  |
| TXNRD1 | rs11111979 | G G | 26 / 26 | 1.00 |  | 0.459 | 1.00 | 1.000 |  |
|  |  | G C | 50 / 43 | 1.11 (0.58; 2.16) | 0.748 |  | 1.00 (0.53; 1.89) |  |  |
|  |  | C C | 15 / 22 | 0.70 (0.30; 1.64) | 0.415 |  |  |  |  |
| TXNRD1 | rs11112011 | C C | 81 / 79 | 1.00 |  | 0.515 | 1.00 | 0.638 |  |
|  |  | C A | 10 / 11 | 0.89 (0.34; 2.30) | 0.809 |  | 0.80 (0.32; 2.03) |  |  |
|  |  | A A | 0 / 1 | 0.00 (0.00; .) | 0.994 |  |  |  |  |
| TXNRD1 | rs11610799 | G G | 77 / 77 | 1.00 |  | 0.695 | 1.00 | 1.000 |  |
|  |  | G C | 14 / 12 | 1.10 (0.47; 2.59) | 0.827 |  | 1.00 (0.43; 2.31) |  |  |
|  |  | C C | 0 / 2 | 0.00 (0.00; .) | 0.992 |  |  |  |  |
| TXNRD1 | rs12810189 | G G | 80 / 80 | 1.00 |  | 1.000 | 1.00 | 1.000 |  |
|  |  | A A | 11 / 11 | 1.00 (0.42; 2.40) | 1.000 |  |  |  |  |
| TXNRD1 | rs17202060 | G G | 40 / 44 | 1.00 |  | 0.514 | 1.00 | 0.547 |  |
|  |  | G A | 39 / 37 | 1.17 (0.62; 2.19) | 0.631 |  | 1.20 (0.66; 2.17) |  |  |
|  |  | A A | 12 / 10 | 1.32 (0.52; 3.37) | 0.561 |  |  |  |  |
| TXNRD1 | rs17808695 | A A | 68 / 57 | 1.00 |  | 0.038 | 1.00 | 0.068 | * |
|  |  | A G | 21 / 27 | 0.60 (0.29; 1.24) | 0.169 |  | 0.52 (0.26; 1.05) |  |  |
|  |  | G G | 2 / 7 | 0.24 (0.05; 1.18) | 0.079 |  |  |  | * |
| TXNRD1 | rs4246269 | A A | 73 / 76 | 1.00 |  | 0.591 | 1.00 | 0.550 |  |
|  |  | A G | 17 / 14 | 1.30 (0.57; 2.97) | 0.533 |  | 1.27 (0.58; 2.80) |  |  |
|  |  | G G | 1 / 1 | 1.00 (0.06; 15.99) | 1.000 |  |  |  |  |
| TXNRD1 | rs4964287 | G G | 45 / 41 | 1.00 |  | 0.426 | 1.00 | 0.538 |  |
|  |  | G A | 37 / 38 | 0.86 (0.46; 1.61) | 0.634 |  | 0.83 (0.45; 1.52) |  |  |
|  |  | A A | 9 / 12 | 0.68 (0.26; 1.77) | 0.427 |  |  |  |  |
| TXNRD1 | rs4964778 | G G | 62 / 64 | 1.00 |  | 0.786 | 1.00 | 0.752 |  |
|  |  | G C | 26 / 24 | 1.11 (0.59; 2.12) | 0.744 |  | 1.11 (0.59; 2.06) |  |  |
|  |  | C C | 3 / 3 | 1.04 (0.21; 5.21) | 0.965 |  |  |  |  |
| TXNRD1 | rs7132880 | A A | 64 / 56 | 1.00 |  | 0.115 | 1.00 | 0.186 |  |
|  |  | A G | 25 / 29 | 0.72 (0.36; 1.47) | 0.372 |  | 0.64 (0.33; 1.24) |  |  |
|  |  | G G | 2 / 6 | 0.31 (0.06; 1.54) | 0.151 |  |  |  |  |
| TXNRD1 | rs7297209 | G G | 47 / 45 | 1.00 |  | 0.416 | 1.00 | 0.746 |  |
|  |  | G A | 28 / 24 | 1.09 (0.53; 2.23) | 0.824 |  | 0.90 (0.48; 1.70) |  |  |
|  |  | A A | 12 / 18 | 0.67 (0.29; 1.54) | 0.347 |  |  |  |  |
| TXNRD1 | rs7297466 | G G | 75 / 68 | 1.00 |  | 0.177 | 1.00 | 0.213 |  |
|  |  | G A | 15 / 20 | 0.69 (0.32; 1.48) | 0.339 |  | 0.63 (0.31; 1.30) |  |  |
|  |  | A A | 1 / 3 | 0.33 (0.04; 3.21) | 0.341 |  |  |  |  |
| TXNRD1 | rs7310814 | C C | 50 / 48 | 1.00 |  | 0.249 | 1.00 | 0.752 |  |
|  |  | C A | 37 / 31 | 1.16 (0.59; 2.28) | 0.668 |  | 0.91 (0.49; 1.68) |  |  |
|  |  | A A | 4 / 12 | 0.36 (0.11; 1.14) | 0.082 |  |  |  | * |
| TXNRD1 | rs7311851 | C C | 22 / 27 | 1.00 |  | 0.833 | 1.00 | 0.413 |  |
|  |  | C A | 49 / 41 | 1.40 (0.71; 2.74) | 0.329 |  | 1.31 (0.69; 2.52) |  |  |
|  |  | A A | 20 / 23 | 1.08 (0.47; 2.48) | 0.857 |  |  |  |  |
| TXNRD1 | rs7953266 | A A | 31 / 29 | 1.00 |  | 0.363 | 1.00 | 0.763 |  |
|  |  | A G | 48 / 43 | 1.05 (0.56; 1.97) | 0.885 |  | 0.91 (0.51; 1.65) |  |  |
|  |  | G G | 12 / 19 | 0.63 (0.27; 1.45) | 0.275 |  |  |  |  |
| TXNRD1 | rs7962759 | C C | 53 / 54 | 1.00 |  | 0.914 | 1.00 | 0.889 |  |
|  |  | C G | 31 / 28 | 1.12 (0.62; 2.02) | 0.714 |  | 1.04 (0.60; 1.80) |  |  |
|  |  | G G | 6 / 8 | 0.77 (0.27; 2.26) | 0.640 |  |  |  |  |
| TXNRD1 | rs7962942 | G G | 55 / 52 | 1.00 |  | 0.462 | 1.00 | 0.648 |  |
|  |  | G A | 32 / 32 | 0.93 (0.50; 1.74) | 0.828 |  | 0.87 (0.48; 1.58) |  |  |
|  |  | A A | 4 / 7 | 0.55 (0.16; 1.96) | 0.358 |  |  |  |  |
| TXNRD1 | rs7977617 | A A | 65 / 66 | 1.00 |  | 0.648 | 1.00 | 0.866 |  |
|  |  | A C | 23 / 24 | 1.00 (0.51; 1.96) | 1.000 |  | 1.06 (0.55; 2.05) |  |  |
|  |  | C C | 3 / 1 | 3.00 (0.30; 29.57) | 0.347 |  |  |  |  |
| TXNRD2 | rs1012157 | G G | 58 / 61 | 1.00 |  | 0.427 | 1.00 | 0.662 |  |
|  |  | G A | 24 / 25 | 1.00 (0.54; 1.86) | 1.000 |  | 1.14 (0.64; 2.02) |  |  |
|  |  | A A | 9 / 5 | 2.00 (0.60; 6.71) | 0.262 |  |  |  |  |
| TXNRD2 | rs1015471 | G G | 53 / 44 | 1.00 |  | 0.320 | 1.00 | 0.210 |  |
|  |  | G A | 27 / 35 | 0.67 (0.36; 1.24) | 0.200 |  | 0.70 (0.40; 1.22) |  |  |
|  |  | A A | 10 / 11 | 0.79 (0.33; 1.92) | 0.609 |  |  |  |  |
| TXNRD2 | rs13057441 | G G | 79 / 81 | 1.00 |  | 0.655 | 1.00 | 0.655 |  |
|  |  | G A | 12 / 10 | 1.22 (0.51; 2.95) | 0.655 |  | 1.22 (0.51; 2.95) |  |  |
| TXNRD2 | rs1544325 | G G | 36 / 42 | 1.00 |  | 0.303 | 1.00 | 0.378 |  |
|  |  | G A | 35 / 34 | 1.19 (0.62; 2.31) | 0.603 |  | 1.30 (0.73; 2.33) |  |  |
|  |  | A A | 19 / 14 | 1.49 (0.69; 3.21) | 0.308 |  |  |  |  |
| TXNRD2 | rs17745314 | G G | 74 / 72 | 1.00 |  | 0.848 | 1.00 | 0.683 |  |
|  |  | G A | 16 / 19 | 0.77 (0.34; 1.75) | 0.533 |  | 0.85 (0.38; 1.89) |  |  |
|  |  | A A | 1 / 0 | 4004360.21 (0.00; .) | 0.994 |  |  |  |  |
| TXNRD2 | rs17745433 | A A | 71 / 64 | 1.00 |  | 0.300 | 1.00 | 0.265 |  |
|  |  | A G | 19 / 26 | 0.68 (0.35; 1.31) | 0.253 |  | 0.70 (0.37; 1.32) |  |  |
|  |  | G G | 1 / 1 | 1.00 (0.06; 15.99) | 1.000 |  |  |  |  |
| TXNRD2 | rs1978058 | G G | 36 / 30 | 1.00 |  | 0.219 | 1.00 | 0.356 |  |
|  |  | G A | 42 / 42 | 0.83 (0.43; 1.59) | 0.571 |  | 0.75 (0.41; 1.38) |  |  |
|  |  | A A | 13 / 19 | 0.58 (0.25; 1.36) | 0.209 |  |  |  |  |
| TXNRD2 | rs2073750 | G G | 54 / 62 | 1.00 |  | 0.407 | 1.00 | 0.220 |  |
|  |  | G A | 36 / 26 | 1.63 (0.86; 3.10) | 0.135 |  | 1.47 (0.79; 2.72) |  |  |
|  |  | A A | 1 / 3 | 0.37 (0.04; 3.63) | 0.395 |  |  |  |  |
| TXNRD2 | rs3747067 | C C | 32 / 24 | 1.00 |  | 0.487 | 1.00 | 0.198 |  |
|  |  | C G | 34 / 43 | 0.60 (0.30; 1.21) | 0.152 |  | 0.65 (0.34; 1.25) |  |  |
|  |  | G G | 25 / 24 | 0.76 (0.34; 1.68) | 0.495 |  |  |  |  |
| TXNRD2 | rs3788305 | A A | 31 / 25 | 1.00 |  | 0.320 | 1.00 | 0.332 |  |
|  |  | A G | 41 / 43 | 0.76 (0.38; 1.56) | 0.459 |  | 0.73 (0.38; 1.39) |  |  |
|  |  | G G | 18 / 22 | 0.67 (0.30; 1.50) | 0.332 |  |  |  |  |
| TXNRD2 | rs3788306 | A A | 47 / 39 | 1.00 |  | 0.219 | 1.00 | 0.241 |  |
|  |  | A G | 36 / 41 | 0.73 (0.39; 1.37) | 0.332 |  | 0.70 (0.39; 1.27) |  |  |
|  |  | G G | 7 / 10 | 0.59 (0.20; 1.71) | 0.330 |  |  |  |  |
| TXNRD2 | rs3788310 | G G | 80 / 69 | 1.00 |  | 0.086 | 1.00 | 0.047 | * |
|  |  | G A | 9 / 20 | 0.42 (0.18; 0.96) | 0.039 |  | 0.45 (0.21; 0.99) |  | * |
|  |  | A A | 2 / 2 | 0.81 (0.11; 5.99) | 0.835 |  |  |  |  |
| TXNRD2 | rs3788314 | G G | 37 / 27 | 1.00 |  | 0.154 | 1.00 | 0.135 |  |
|  |  | G A | 33 / 37 | 0.66 (0.34; 1.29) | 0.222 |  | 0.63 (0.34; 1.16) |  |  |
|  |  | A A | 21 / 27 | 0.59 (0.29; 1.22) | 0.158 |  |  |  |  |
| TXNRD2 | rs3788317 | C C | 61 / 52 | 1.00 |  | 0.245 | 1.00 | 0.173 |  |
|  |  | C A | 27 / 36 | 0.62 (0.33; 1.19) | 0.152 |  | 0.65 (0.36; 1.21) |  |  |
|  |  | A A | 3 / 3 | 0.93 (0.19; 4.63) | 0.925 |  |  |  |  |
| TXNRD2 | rs4333017 | G G | 47 / 39 | 1.00 |  | 0.367 | 1.00 | 0.209 |  |
|  |  | G A | 35 / 43 | 0.62 (0.31; 1.24) | 0.177 |  | 0.67 (0.35; 1.26) |  |  |
|  |  | A A | 9 / 9 | 0.81 (0.31; 2.16) | 0.675 |  |  |  |  |
| TXNRD2 | rs4485648 | A A | 56 / 49 | 1.00 |  | 0.401 | 1.00 | 0.277 |  |
|  |  | A G | 27 / 34 | 0.67 (0.34; 1.31) | 0.244 |  | 0.71 (0.38; 1.32) |  |  |
|  |  | G G | 8 / 8 | 0.88 (0.28; 2.79) | 0.823 |  |  |  |  |
| TXNRD2 | rs5992493 | A A | 68 / 59 | 1.00 |  | 0.157 | 1.00 | 0.192 |  |
|  |  | A G | 22 / 29 | 0.72 (0.39; 1.32) | 0.288 |  | 0.68 (0.38; 1.22) |  |  |
|  |  | G G | 1 / 3 | 0.33 (0.04; 3.21) | 0.341 |  |  |  |  |
| TXNRD2 | rs7288061 | G G | 63 / 64 | 1.00 |  | 0.882 | 1.00 | 0.873 |  |
|  |  | G A | 27 / 26 | 1.06 (0.55; 2.01) | 0.870 |  | 1.05 (0.56; 1.97) |  |  |
|  |  | A A | 1 / 1 | 1.00 (0.06; 15.99) | 1.000 |  |  |  |  |
| TXNRD2 | rs7289747 | A A | 79 / 84 | 1.00 |  | 0.232 | 1.00 | 0.232 |  |
|  |  | A C | 12 / 7 | 1.83 (0.68; 4.96) | 0.232 |  | 1.83 (0.68; 4.96) |  |  |
| TXNRD2 | rs737854 | G G | 68 / 71 | 1.00 |  | 0.758 | 1.00 | 0.602 |  |
|  |  | G A | 20 / 16 | 1.31 (0.64; 2.69) | 0.467 |  | 1.20 (0.61; 2.38) |  |  |
|  |  | A A | 1 / 2 | 0.50 (0.05; 5.51) | 0.572 |  |  |  |  |
| TXNRD2 | rs7410379 | G G | 48 / 49 | 1.00 |  | 0.663 | 1.00 | 0.884 |  |
|  |  | G A | 32 / 34 | 0.96 (0.52; 1.78) | 0.905 |  | 1.04 (0.59; 1.85) |  |  |
|  |  | A A | 11 / 8 | 1.41 (0.52; 3.82) | 0.503 |  |  |  |  |
| TXNRD2 | rs8141691 | G G | 48 / 43 | 1.00 |  | 0.913 | 1.00 | 0.457 |  |
|  |  | G A | 31 / 40 | 0.69 (0.37; 1.30) | 0.251 |  | 0.80 (0.44; 1.44) |  |  |
|  |  | A A | 12 / 8 | 1.34 (0.50; 3.59) | 0.565 |  |  |  |  |
| TXNRD2 | rs933271 | A A | 44 / 40 | 1.00 |  | 0.768 | 1.00 | 0.580 |  |
|  |  | A G | 32 / 37 | 0.81 (0.45; 1.47) | 0.485 |  | 0.86 (0.50; 1.48) |  |  |
|  |  | G G | 15 / 14 | 1.00 (0.43; 2.35) | 0.993 |  |  |  |  |
| TXNRD2 | rs9605030 | G G | 67 / 66 | 1.00 |  | 0.662 | 1.00 | 0.873 |  |
|  |  | G A | 23 / 22 | 1.00 (0.53; 1.89) | 1.000 |  | 0.95 (0.51; 1.78) |  |  |
|  |  | A A | 1 / 3 | 0.33 (0.03; 3.28) | 0.346 |  |  |  |  |
| TXNRD2 | rs9605031 | G G | 52 / 50 | 1.00 |  | 0.464 | 1.00 | 0.768 |  |
|  |  | G A | 31 / 28 | 1.05 (0.56; 1.96) | 0.891 |  | 0.92 (0.51; 1.64) |  |  |
|  |  | A A | 8 / 13 | 0.63 (0.25; 1.58) | 0.321 |  |  |  |  |
| TXNRD2 | rs9606173 | A A | 71 / 59 | 1.00 |  | 0.094 | 1.00 | 0.068 | * |
|  |  | A T | 18 / 30 | 0.55 (0.29; 1.04) | 0.065 |  | 0.56 (0.30; 1.04) |  | * |
|  |  | T T | 2 / 2 | 0.74 (0.10; 5.51) | 0.769 |  |  |  |  |
| TXNRD2 | rs9606174 | A A | 78 / 69 | 1.00 |  | 0.166 | 1.00 | 0.111 |  |
|  |  | A G | 11 / 20 | 0.52 (0.24; 1.13) | 0.099 |  | 0.55 (0.26; 1.15) |  | * |
|  |  | G G | 2 / 2 | 0.85 (0.12; 6.22) | 0.875 |  |  |  |  |
| TXNRD2 | rs9606176 | G G | 80 / 72 | 1.00 |  | 0.178 | 1.00 | 0.110 |  |
|  |  | G A | 8 / 16 | 0.46 (0.19; 1.14) | 0.094 |  | 0.50 (0.21; 1.17) |  | * |
|  |  | A A | 2 / 2 | 0.83 (0.11; 6.10) | 0.853 |  |  |  |  |
| USP4 | rs9818758 | G G | 89 / 89 | 1.00 |  |  | 1.00 |  |  |
| YBX1 | rs10493112 | C C | 26 / 19 | 1.00 |  | 0.449 | 1.00 | 0.227 |  |
|  |  | C A | 44 / 51 | 0.63 (0.30; 1.30) | 0.209 |  | 0.65 (0.32; 1.31) |  |  |
|  |  | A A | 21 / 21 | 0.71 (0.30; 1.68) | 0.437 |  |  |  |  |
| YBX1 | rs10493113 | G G | 68 / 72 | 1.00 |  | 0.400 | 1.00 | 0.481 |  |
|  |  | G A | 22 / 19 | 1.21 (0.60; 2.46) | 0.591 |  | 1.29 (0.64; 2.59) |  |  |
|  |  | A A | 1 / 0 | 4004360.21 (0.00; .) | 0.994 |  |  |  |  |
| YBX1 | rs11210696 | C C | 38 / 32 | 1.00 |  | 0.225 | 1.00 | 0.367 |  |
|  |  | C A | 44 / 45 | 0.82 (0.44; 1.52) | 0.521 |  | 0.76 (0.42; 1.38) |  |  |
|  |  | A A | 9 / 14 | 0.54 (0.21; 1.42) | 0.214 |  |  |  |  |
| YBX1 | rs11806746 | C C | 38 / 39 | 1.00 |  | 0.715 | 1.00 | 0.882 |  |
|  |  | C A | 44 / 45 | 1.01 (0.55; 1.83) | 0.980 |  | 1.05 (0.58; 1.88) |  |  |
|  |  | A A | 9 / 7 | 1.41 (0.42; 4.67) | 0.577 |  |  |  |  |
| YBX1 | rs11809002 | G G | 85 / 86 | 1.00 |  | 0.413 | 1.00 | 0.739 |  |
|  |  | G A | 4 / 5 | 1.00 (0.25; 4.00) | 1.000 |  | 1.25 (0.34; 4.66) |  |  |
|  |  | A A | 2 / 0 | 4004360.21 (0.00; .) | 0.991 |  |  |  |  |
| YBX1 | rs12030724 | A A | 70 / 74 | 1.00 |  | 0.506 | 1.00 | 0.481 |  |
|  |  | A T | 20 / 16 | 1.29 (0.64; 2.61) | 0.477 |  | 1.29 (0.64; 2.59) |  |  |
|  |  | T T | 1 / 1 | 1.14 (0.07; 18.68) | 0.929 |  |  |  |  |
| YBX1 | rs6670466 | A A | 43 / 35 | 1.00 |  | 0.064 | 1.00 | 0.230 | * |
|  |  | A G | 43 / 43 | 0.78 (0.42; 1.45) | 0.435 |  | 0.69 (0.38; 1.26) |  |  |
|  |  | G G | 5 / 13 | 0.29 (0.09; 0.95) | 0.041 |  |  |  | * |
| ZDHHC5 | rs11604333 | G G | 81 / 79 | 1.00 |  | 0.722 | 1.00 | 0.655 |  |
|  |  | G C | 1 / 2 | 0.50 (0.05; 5.51) | 0.572 |  | 0.82 (0.34; 1.97) |  |  |
|  |  | C C | 8 / 9 | 0.89 (0.34; 2.30) | 0.809 |  |  |  |  |
| ZDHHC5 | rs12801694 | G G | 51 / 48 | 1.00 |  | 0.584 | 1.00 | 0.681 |  |
|  |  | G A | 34 / 35 | 0.94 (0.53; 1.67) | 0.825 |  | 0.89 (0.52; 1.53) |  |  |
|  |  | A A | 6 / 8 | 0.70 (0.22; 2.24) | 0.550 |  |  |  |  |
